# Hemispheric Contributions to Figurative Language Comprehension After Unilateral Brain Damage: A Meta‐Analysis of Lesion Side and Conventionality Interaction

**DOI:** 10.1002/brb3.71828

**Published:** 2026-10-07

**Authors:** Lexian Shen, Man Fu, Min Wang, Xuemei Tang, Hui Chang

**Affiliations:** ^1^ School of Foreign Languages Shanghai Jiao Tong University Shanghai China; ^2^ National Research Center For Language and Well‐Being Shanghai Jiao Tong University Shanghai China; ^3^ School of Foreign Studies Anhui Polytechnic University Wuhu China; ^4^ Language Disorder and Brain Science Research Center Hunan Normal University Hunan China

**Keywords:** brain damage, figurative language, lateralization, meta‐analysis

## Abstract

**Introduction:**

Previous studies revealed that individuals with brain damage exhibit distinctive patterns in figurative language comprehension. However, a systematic investigation of the mechanisms between figurative language deficits and moderating factors such as lesion side remains lacking. Moreover, while the right hemisphere has traditionally been emphasized in pragmatic processing, accumulating evidence now suggests that the contributions of the left and right hemispheres may depend on linguistic factors such as conventionality. A recent systematic review on idiom comprehension in aphasia has highlighted the critical role of stimulus characteristics, yet no quantitative synthesis has examined how conventionality modulates hemispheric contributions to figurative language processing after unilateral brain damage. This meta‐analysis aims to: (1) quantify the overall difference between left‐ and right‐hemisphere damaged (LBD and RBD) patients in figurative language comprehension accuracy; (2) examine whether conventionality moderates hemispheric differences; (3) explore potential moderators—including aphasia status, task type, and sample size—that may influence the LBD‐RBD difference; and (4) compare impairment patterns of LBD and RBD patients relative to healthy controls across different conventionality levels.

**Methods:**

Following PRISMA guidelines, a systematic literature search was conducted across Web of Science, PubMed, and ProQuest. Studies comparing LBD and/or RBD patients to healthy controls on accuracy in figurative language comprehension tasks were included. Multi‐level meta‐analyses were performed to estimate overall effect sizes, examine moderating effects of conventionality, and compare impairment patterns. Meta‐regressions explored potential moderators including aphasia status, sample size, task types, and number of trials.

**Results:**

Forty‐five articles met inclusion criteria, with 13 articles providing direct LBD‐RBD comparisons (516 LBD; 476 RBD patients). Overall, no significant main effect of hemisphere emerged. Although the interaction between conventionality and lesion side did not reach statistical significance, the pattern of effect sizes across conventionality levels revealed meaningful trends. At high conventionality, LBD patients showed greater deficits than RBD patients, while at low conventionality, RBD patients showed greater deficits. Moderator analyses further revealed that task type and sample size significantly modulated the overall LBD‐RBD difference, whereas aphasia status and number of trials did not. Additional comparisons including 70 LBD versus HC studies (1120 LBD patients, 1342 HC) and 68 RBD versus HC studies (1135 RBD patients, 1291 HC) confirmed that both patient groups are significantly impaired relative to healthy controls across all conventionality levels.

**Conclusion:**

Hemispheric contributions to figurative language comprehension are dynamic and conventionality‐dependent. The left hemisphere is particularly crucial for highly conventionalized expressions, while both hemispheres contribute to novel expressions. These findings are further qualified by methodological moderators: task type and sample size significantly modulated the observed hemispheric differences, whereas aphasia status and number of trials did not. The results have implications for neurocognitive models of figurative language and for the design of clinical assessments, highlighting the need to consider both conventionality and task demands when evaluating figurative language abilities in brain‐damaged populations.

## 1 Introduction

While early clinical observations by Critchley (1962) and experimental work by Eisenson (1962) first suggested a link between right hemisphere brain damage and deficits in abstract language use, Gardner et al. (1975) were among the first to conduct systematic experimental investigations specifically targeting the comprehension of figurative language, such as metaphors. Following a series of psychological experiments (Winner and Gardner 1977), they observed distinct patterns of impairment in comprehending metaphors between patients with left and right hemisphere damage, prompting increasing scholarly attention to this topic. Subsequent studies have examined various types of figurative language, including conventional metaphors (e.g., Bryan 1988; Giora et al. 2000), humor (Zaidel et al. 2002), irony (Shamay‐Tsoory et al. 2005), idioms (Brundage 1993; Aydin et al. 2017), and proverbs (England 1986). Some researchers have further attempted to incorporate figurative language into clinical assessment batteries for special populations such as patients with aphasia (e.g., Brownell et al. 2007; Siqueira and Marques 2018) or as therapeutic tools (Lundgren et al. 2011; Tompkins et al. 2012). Supporting information

However, findings regarding the patterns of figurative language comprehension deficits after brain damage remain inconsistent across studies. Compared to research with healthy adults, the variables examined and controlled for in patient studies are still insufficient. For instance, evidence from healthy adult studies, particularly cognitive neuroscience findings such as those reported by Tang et al. (2017), suggests that conventionality is a critical variable in figurative language processing (Bowdle and Gentner 2005). To date, several studies have independently examined figurative language with different levels of conventionality, broadly covering all three levels (high: idioms, proverbs, and frozen/dead metaphors; medium: conventional metaphors and puns; low: novel metaphors, irony, and humor). Nevertheless, no study has specifically investigated how conventionality modulates the pattern of figurative language comprehension deficits in brain‐damaged populations, nor has any systematic review or meta‐analysis synthesized findings across these diverse experimental studies.

### 1.1 Figurative Language Comprehension: Evidence From Healthy and Brain‐Damaged Populations

In healthy adults, figurative language comprehension has been characterized as a complex cognitive process involving both automatic and controlled mechanisms. Some theories emphasize the automatic nature of metaphor processing, particularly for highly conventionalized expressions. According to the Conceptual Metaphor Theory (Lakoff and Johnson 1980), the understanding of conventional metaphors such as “*Argument is war*” involves automatically activating pre‐existing conceptual mappings stored in long‐term memory, requiring minimal cognitive effort. This automaticity is thought to arise from repeated co‐activation of source and target domains, which strengthens the neural connections between them over time.

In contrast, other theoretical frameworks highlight the complex, effortful processes involved in comprehending less conventionalized figurative expressions. The Structure Mapping Theory (Gentner and Markman 1997; Wolff and Gentner 2011) proposes that metaphor comprehension involves an active process of aligning the relational structures of source and target domains and a later directional mapping on these aligned structures from the source domains to the target domains. Unlike simple property attribution, this view suggests that understanding a novel metaphor such as “A rumour is a weed” requires identifying systematic correspondences between the two domains—for example, both rumours and weeds spread rapidly, are difficult to control once established, and can cause damage to their surroundings. This structural alignment process is resource‐demanding and recruits executive control mechanisms to inhibit irrelevant features while maintaining relevant relational structures in working memory (Gentner and Wolff 1997).

The apparent contradiction between these perspectives has been reconciled by the career of metaphor (Bowdle and Gentner 2005), which proposes that the processing mechanism recruited for metaphor comprehension depends critically on the degree of conventionality. According to this hypothesis, novel metaphors are processed via structural mapping, requiring active comparison and inference to establish new cross‐domain mapping. As metaphors become increasingly conventionalized through repeated use, they undergo a process of abstraction, eventually becoming lexicalized as new categorical meanings. Highly conventional metaphors, like dead metaphors, are then processed via category‐based retrieval, similar to literal language. This theoretical framework predicts that conventionality should systematically modulate the cognitive and neural resources engaged during figurative language processing, which has received substantial empirical support from studies with healthy adults (e.g., Tang et al. 2017; Lai et al. 2009).

Turning to brain‐damaged populations, existing research has demonstrated that individuals with brain damage exhibit pervasive deficits in figurative language comprehension, regardless of the lesion side (e.g., Bryan 1988; Papagno et al. 2004; Winner and Gardner 1977). However, the severity and pattern of these deficits are modulated by multiple factors, among which the lesion side (left vs. right hemisphere damage) emerges as a critical determinant (e.g., Van Lancker and Kempler 1987; Brownell et al. 1990). Understanding how lesion side interacts with figurative language processing is essential for developing comprehensive models of hemispheric contributions to figurative language comprehension.

### 1.2 Lateralization Hypotheses on Figurative Language Processing

It is well established that the left hemisphere (LH) is generally dominant for most core linguistic processes, such as syntax and lexical semantics (Friederici and Alter 2004). Building on this foundation, several theoretical frameworks have been proposed to account for hemispheric asymmetries in the processing of figurative language specifically. Among the most influential is the fine‐coarse semantic coding theory (Jung‐Beeman 2005), which proposed a framework for the different roles of the left hemisphere and the right hemisphere (RH) in semantic activation, integration, and selection. According to this framework, the LH engages in “fine semantic coding”: It rapidly activates a narrow set of closely related or contextually relevant meanings, enabling efficient and precise semantic selection. The RH, in contrast, engages in “coarser semantic coding”: It maintains diffuse activation across a broader semantic field, including distant or weakly related concepts, which facilitates the integration of novel or non‐literal meanings.

Under this framework, the processing of figurative language, especially that with low conventionality such as novel metaphors, humors, and ironies, including not only the semantic activation of a relatively broader semantic field, but also a further stage of complex semantic integration, relies on the involvement of the right hemisphere. This pattern of increased RH involvement in novel metaphor comprehension has been found in several neuroimaging studies (Mashal et al. 2007; Schmidt et al. 2007). The LH, in contrast, which can quickly focus semantic activation and selection on literal or contextually relevant meaning, may also have an advantage in the processing of figurative language with high conventionality such as idioms, dead metaphors, and even conventional metaphors, since the figurative meaning of a conventional metaphor has established a relatively fixed connection with its expression according to the career of metaphor (Bowdle and Gentner 2005). Similarly, the relatively greater involvement of LH compared to RH in conventional metaphor comprehension has also been found in neurolinguistic studies (Huang et al. 2022; Rapp et al. 2007).

However, empirical findings have not been entirely consistent with coarse coding predictions. Using visual half‐field priming paradigms, Kandhadai and Federmeier (2007) found no hemispheric differences in summation priming across mediated primes or divergent meanings of ambiguous words. Similarly, Mech et al. (2022), using ERPs to distinguish automatic (N400) from controlled (LPC) semantic processing, systematically manipulated relation type (associative vs. categorical) and strength (strong vs. weak), and found nearly identical priming patterns across hemispheres, with no evidence for an RH advantage for weak or categorical relations, suggesting that task and context may shape the way each hemisphere utilizes semantic information in service of the task goal, rather than revealing fundamental representational differences between the two hemispheres.

Consistent with these theoretical predictions, studies comparing patients with left‐ and right‐hemisphere damage have revealed distinct patterns of impairment that vary as a function of conventionality. Some studies have reported that patients with left‐hemisphere damage show greater deficits than those with right‐hemisphere damage for high‐conventionality expressions such as proverbs (e.g., England 1986; Ulatowska et al. 2000) and conventional metaphors (e.g., Bryan 1988; Giora et al. 2000). In contrast, other investigations have found the opposite pattern for low‐conventionality expressions, with right‐hemisphere damaged patients exhibiting more severe impairments in comprehending novel metaphors (e.g., Brownell et al. 1990; Winner and Gardner 1977) and irony (e.g., Shamay‐Tsoory et al. 2005; Zaidel et al. 2002). The picture becomes even more complex for medium‐conventionality expressions such as conventional metaphors and puns, where findings are particularly inconsistent and appear to be modulated by additional factors such as aphasia status within the left‐hemisphere damaged group (e.g., Papagno and Cacciari 2010; Cacciari et al. 2006). This heterogeneity across studies underscores the need for a quantitative synthesis that can systematically examine how conventionality moderates hemispheric contributions to figurative language comprehension.

### 1.3 Rationale for an Updated Review

Despite decades of research on figurative language comprehension in brain‐damaged populations, several critical gaps remain. First, while previous studies have independently examined various types of figurative expressions—including idioms, proverbs, conventional metaphors, novel metaphors, irony, and humor—no study has systematically investigated how the degree of conventionality modulates the pattern of deficits in patients with left‐ versus right‐hemisphere damage. The career of metaphor (Bowdle and Gentner 2005) and the fine‐coarse semantic coding theory (Jung‐Beeman 2005) both predict that conventionality should be a critical determinant of hemispheric contributions, yet this prediction has limited empirical investigation in patient populations.

Second, existing findings are highly heterogeneous, with some studies reporting greater deficits in left‐hemisphere damaged patients for certain types of figurative language and others reporting the opposite pattern for other types. This inconsistency may reflect methodological differences across studies, including variations in stimulus materials, task demands, and patient characteristics such as aphasia status. A quantitative synthesis is needed to determine whether these apparent discrepancies reflect genuine differences in hemispheric specialization or are merely artifacts of methodological heterogeneity.

Third, although a recent systematic review has examined idiom comprehension in patients with aphasia (Lada et al. 2023), this review focused exclusively on idioms and did not provide a quantitative synthesis of effect sizes across studies. Moreover, no review to date has systematically compared patterns of impairment across different levels of conventionality within the same analytic framework, nor has any study meta‐analytically examined the role of aphasia status in modulating hemispheric contributions to figurative language comprehension.

To address these gaps, the present meta‐analysis aims to: (1) quantify the overall difference between left‐ and right‐hemisphere damaged patients in figurative language comprehension accuracy; (2) examine whether the degree of conventionality (high, medium, and low) moderates the pattern of hemispheric differences; (3) investigate the role of aphasia status in left‐hemisphere damaged patients as a potential moderator; and (4) compare the impairment patterns of left‐ and right‐hemisphere damaged patients relative to healthy controls across different levels of conventionality.

## 2 Method

### 2.1 Identification of Relevant Studies

The review adheres to the guidelines provided by the Preferred Reporting Items for Systematic Reviews and Meta‐Analyses [PRISMA] (Page et al. 2021). A literature search was conducted in the Web of Science Core Collection, PubMed, and ProQuest for relevant articles. The search string used in Web of Science and PubMed was: ((“aphas*”) OR (((“left hemisphere” OR “right hemisphere”) AND (“damage” OR “lesion” OR “injury”)))) AND ((“idiom” OR “proverb” OR “metaphor” OR “pun*” OR “irony” OR “humor*” OR “figurative language”)) AND ((“comprehension” OR “understanding” OR “processing” OR “interpretation”)). For ProQuest, the search was restricted to the NOFT field (i.e., anywhere except full text) using the same keywords. We also manually examined the reference lists of all articles included in the meta‐analysis and related reviews for potentially relevant articles. The search was done between December 24, 2025, and March 5, 2026. Figure 1 summarizes the literature search process.

**FIGURE 1:**
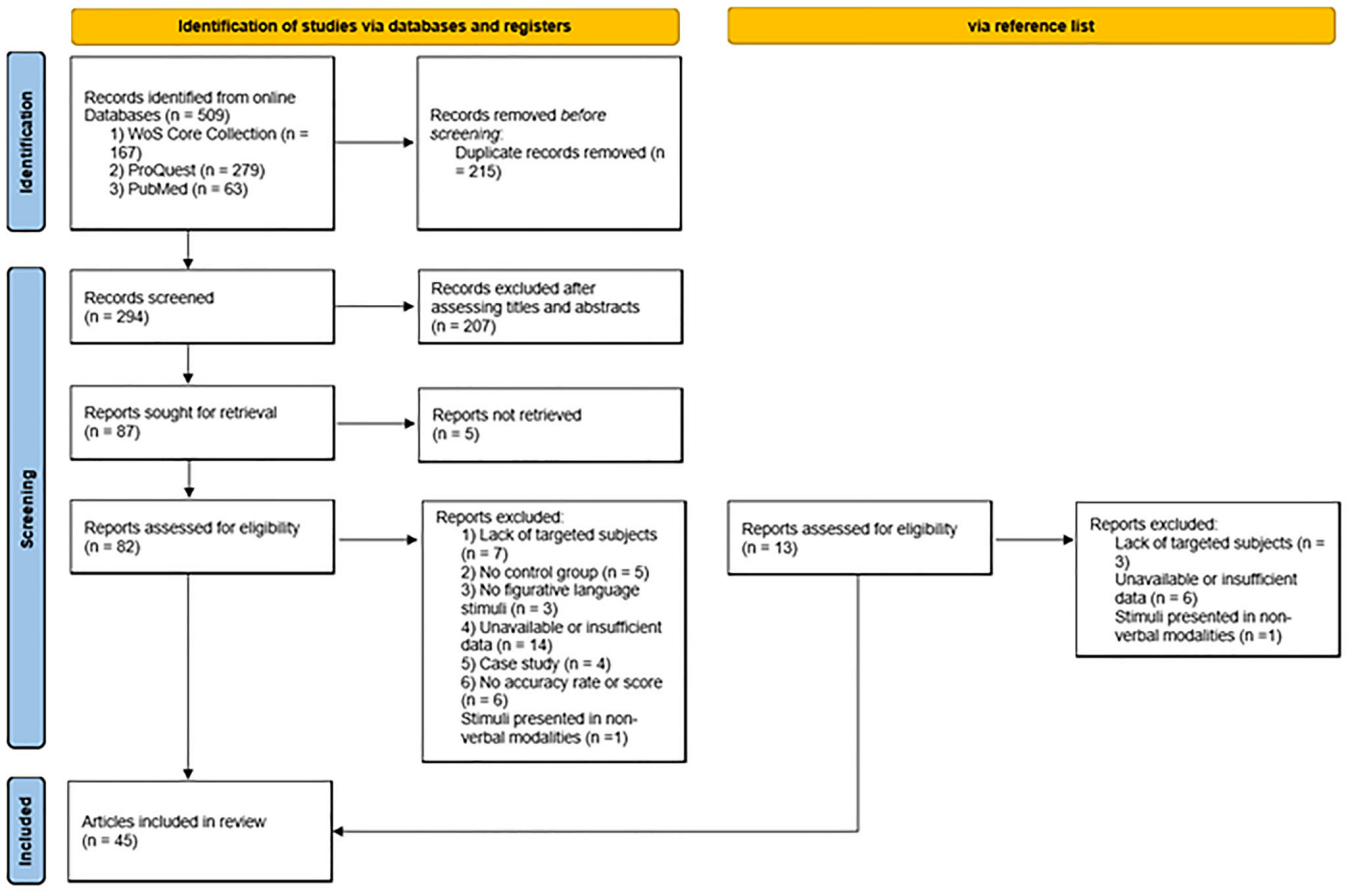
Prisma flow diagram for literature search.

The initial search yielded a total of 509 records (ProQuest: 279; PubMed: 63; Web of Science Core Collection: 167). After removing 215 duplicates, 294 unique records remained for screening. The screening process was conducted independently by two authors (Lexian Shen and Man Fu). Both authors screened all titles and abstracts against the pre‐defined inclusion and exclusion criteria. After completing the initial screening, they compared their decisions and resolved any discrepancies through discussion; when consensus could not be reached, a third author (Min Wang) was consulted to make the final decision. The same procedure was applied during the full‐text screening stage. The titles and abstracts of these 294 records were assessed against the following exclusion criteria: (1) studies not involving individuals with brain damage (defined as acute acquired unilateral focal lesions, excluding progressive neurological conditions and slow‐growing tumors), such as studies with only healthy participants or populations with dementia; (2) non‐empirical studies, such as review articles; (3) studies employing methodologies such as text/conversation analysis or acoustic analysis of production; (4) studies not involving figurative language; (5) studies focusing on assessment or treatment tools without reporting patients’ actual performance data. Following this initial screening, 207 records were excluded, leaving 87 full‐text articles to be retrieved for further assessment. Five of these articles could not be retrieved, resulting in 82 full‐text articles assessed for eligibility. An additional 13 articles identified through reference list screening were also assessed, bringing the total number of full‐text articles assessed for eligibility to 95.

From these 95 full‐text articles, 50 were excluded. The reasons for exclusion were as follows: (1) failure to distinguish or strictly distinguish between patients with left and right hemisphere damage (e.g., Cordonier et al. 2020; studies with bilateral or mixed samples were excluded unless data for unilateral subgroups were separately extractable, as our aim was to isolate the specific contribution of each hemisphere); (2) absence of a comparison group (i.e., either another patient group with damage in the opposite hemisphere or a healthy control group; e.g., Mancopes and Schultz 2008); (3) absence of figurative language stimuli (e.g., Baldo et al. 2016); (4) unavailable or insufficient data, including incomplete descriptive statistics (e.g., missing standard deviations) or imprecise inferential statistics (such as only reporting *p* < 0.05, e.g., Duque et al. 2021); (5) case studies (e.g., Calvo et al. 2019); (6) studies reporting only reaction times or reading times without accuracy data^1^; (7) stimuli presented in non‐verbal modalities (e.g., Dagge and Hartje 1985).

Following the full‐text review, a total of 45 articles met all inclusion criteria and were included in the meta‐analysis.

### 2.2 Data Extraction

Data extraction was performed independently by two authors (Lexian Shen and Man Fu) using a pre‐defined coding sheet. Discrepancies were resolved through discussion or, when necessary, by consulting a third author (Min Wang). The following information was extracted from each included study: (i) study characteristics (authors, publication year, and study ID); (ii) participant characteristics (sample size for LBD [left‐brain‐damaged] patients, RBD [right‐brain‐damaged] patients, and HC [healthy controls]; LBD subtype, i.e., with or without aphasia; lesion region^2^); (iii) demographic and clinical information including age, education, etiology, lesion size, time of assessment, aphasia severity, and comorbid conditions (these variables were extracted for studies included in the direct LBD‐RBD comparison to characterize the patient samples as summarized in Section 3.1); (iv) stimulus characteristics (conventionality level including high, medium, low, and non‐figurative language; figurative language type including idiom, proverb, metaphor, pun, irony, and humor; and stimulus subtype where applicable, e.g., familiar vs. unfamiliar idioms); (v) task characteristics (task type, e.g., multiple picture selection, verbal meaning interpretation, multiple sentence, or word selection, yes–no question; number of trials); and (vi) outcome data, including descriptive statistics (mean accuracy and standard deviation for LBD, RBD, and HC groups) and, where available, inferential statistics comparing patient groups to healthy controls (statistic type including *F*‐test, *t*‐test, or *Z*‐test; statistic value; degrees of freedom; and *p* value). For studies that reported accuracy separately for multiple patient subgroups or stimulus types, data were extracted as independent entries to preserve the granularity of the original findings.

### 2.3 Moderator Variables

Moderator variables were selected based on theoretical considerations and previous empirical findings. Theoretical variables included lesion side (left vs. right hemisphere damage) and conventionality level, which were central to testing the prediction of conventionality dependent hemispheric contribution. Following the classification used in previous research (Bowdle and Gentner 2005) and adapted to the patient literature, conventionality was operationalized as follows: High conventionality included idioms, proverbs, and frozen/dead metaphors; medium conventionality included conventional metaphors and puns; low conventionality included novel metaphors, irony, humor, and unfamiliar idioms as classified by original studies (e.g., Brundage 1993; IITAKA et al. 2003). Non‐figurative/literal language stimuli were included as a baseline condition.

Participant‐related moderators included aphasia status in LBD patients (with aphasia vs. without aphasia), given its potential to modulate figurative language performance as suggested by preliminary evidence. Task‐related moderators included task type, sample size, and number of trials. Demographic variables such as age and education were not included as moderators, as all included studies employed healthy control groups matched to patient groups on these variables. Following the practice of Witteman et al. (2011), moderators were only included in the analysis if reported in at least five studies; this criterion was applied to the selected participant‐ and task‐related moderators listed in Table 1.

**TABLE 1:** Moderator variables, levels, and operational definitions.

Moderator	Levels	Operational definition
*Theoretical variables*		
Lesion side	LBD/RBD	Left‐hemisphere damage vs. right‐hemisphere damage, as confirmed by neuroimaging or surgical reports in original studies
Conventionality	High	Idioms, proverbs, frozen/dead metaphors
	Medium	Conventional metaphors, puns
	Low	Novel metaphors, irony, humor, and unfamiliar idioms as classified by original studies (e.g., Brundage 1993; Iitaka et al. 2003)
	NF (baseline)	Non‐figurative/literal language stimuli
Participant‐related variables		
Aphasia status in LBD patients	With aphasia/without aphasia	Aphasia status in LBD patients as diagnosed using standardized assessments (e.g., WAB and BDAE) in original studies
Sample size	Continuous	Total number of participants in each patient group (LBD and RBD)
*Task‐related variables*		
Task type	Multiple picture selection	Tasks requiring participants to select a picture matching the meaning of a figurative expression
	Multiple sentence/word selection	Tasks requiring participants to select from written words or sentences
	Verbal meaning explanation	Tasks requiring participants to orally explain the meaning of a figurative expression
	Yes–no question	Tasks requiring participants to make a binary judgment (e.g., whether a sentence makes sense, whether two stimuli have similar meanings)
Number of trials	Continuous	Number of experimental trials per condition

### 2.4 Statistical Analysis

A multi‐level random effects meta‐analysis model were conducted using R (version 4.5.2). Hedges’ *g* was adopted as the effect size estimate for accuracy measures, which includes a correction factor for small sample bias (Hedges and Olkin 2014). Effect sizes were interpreted using Cohen's (1988) benchmarks: 0.2 for small effects, 0.5 for medium effects, and 0.8 for large effects.

Heterogeneity was assessed using Cochran's *Q* statistic (Lipsey and Wilson 2001) and the *I* ^2^ statistic (Higgins and Thompson 2002). *I* ^2^ values of 25%, 50%, and 75% were interpreted as low, moderate, and high heterogeneity, respectively (Higgins et al. 2003). Prediction intervals were calculated to estimate the range of true effects that could be expected in future similar studies (Harrer et al. 2021).

Influence analysis was conducted using the leave‐one‐out method to identify potential outliers and assess the robustness of the pooled effect sizes (Viechtbauer and Cheung 2010). Studies were considered outliers if their effect size confidence intervals did not overlap with the confidence interval of the pooled effect.

Publication bias was examined through visual inspection of funnel plots (Lipsey and Wilson 2001) and formally tested using Egger's regression test (Egger et al. 1997). If significant asymmetry was detected (*p* < 0.05), the trim‐and‐fill method (Duval and Tweedie 2000) was applied to impute missing studies and recalculate adjusted pooled effect sizes.

Moderator analyses were conducted using meta‐regression to examine the extent to which participant‐ and task‐related factors explained heterogeneity in effect sizes. For categorical moderators, subgroup analyses were performed; for continuous moderators, meta‐regression models were fitted. Regression coefficients, *Q*_model_ statistics, and associated *p* values were reported. The proportion of between‐study variance explained by each moderator was estimated using the *R* ^2^ statistic (Borenstein et al. 2009).

### 2.5 Quality Assessment

The methodological quality of the included studies was assessed using the standard quality assessment criteria for quantitative studies (Kmet et al. 2004). Given that none of the included studies involved interventions, three items related to intervention and blinding (items 5, 6, and 7) were excluded from the assessment. Quality assessment results are presented in the corresponding tables reported in the Results section.

## 3 Results

Given that the majority of included studies did not include both left‐ and right‐hemisphere damaged patient groups, the present meta‐analysis was conducted in two stages. The first stage focused on studies that directly compared LBD and RBD patients on figurative language comprehension tasks, addressing the two primary research questions: (1) whether LBD and RBD patients show differential deficits, and (2) whether conventionality moderates the pattern of hemispheric contributions. The second stage separately examined LBD versus healthy controls and RBD versus healthy controls to indirectly assess whether the impairment patterns of the two patient groups were consistent across conventionality levels.

### 3.1 Direct Comparisons Between LBD and RBD Patients

A total of 28 studies from 13 articles met the inclusion criteria for the direct comparison between LBD and RBD patients on figurative language comprehension tasks, comprising 516 LBD and 476 RBD participants. In addition, 8 studies from the same articles examined non‐figurative/literal language comprehension, serving as baseline control conditions. The distribution of studies across conventionality levels was as follows: high conventionality (idioms, proverbs), 10 studies; medium conventionality (conventional metaphors, puns), 10 studies; low conventionality (novel metaphors, irony, humor), 8 studies; and non‐figurative/literal language baseline, 8 studies (see Table 2).

**TABLE 2:** Summary of studies included in the direct comparison between LBD and RBD patients.

No	StudyID	QA	Conventionality	FLType	FLSubType	Hedges’ *g* [95% CI]	SampleSize	n.LBD	LBDSubType	n.RBD	Task	n.Trial
1	Aydin et al. (2017)‐1	20	H	Idiom	Transparent	−0.14 [−1, 0.71]	21	11	With aphasia	10	Picture	16
2	Aydin et al. (2017)‐2	20	H	Idiom	Opaque	−2.11 [−3.19, −1.03]	21	11	With aphasia	10	Picture	31
3	Aydin et al. (2017)‐3	20	H	Idiom	Transparent	−0.23 [−1.09, 0.63]	21	11	With aphasia	10	Verbal	16
4	Aydin et al. (2017)‐4	20	H	Idiom	Opaque	−2.14 [−3.23, −1.05]	21	11	With aphasia	10	Verbal	31
5	Brundage (1993)‐1	21	H	Idiom	Familiar	−0.28 [−1.16, 0.6]	20	10	With aphasia	10	Verbal	12
6	England (1986)‐1	20	H	Proverb		−0.36 [−1.07, 0.35]	31	15	With aphasia	16	Verbal	40
7	IITAKA et al. (2003)‐1	20	H	Idiom	Familiar	0.09 [−0.66, 0.85]	36	27	With aphasia	9	Written	10
8	IITAKA et al. (2003)‐2	20	H	Idiom	Familiar	0.78 [0.01, 1.56]	36	27	With aphasia	9	Written	10
9	Murphy et al. (2013)	22	H	Proverb		−0.2 [−0.75, 0.36]	50	27	Without aphasia	23	Verbal	8
10	Kempler et al. (1999)‐1	22	H	Idiom		1.73 [0.99, 2.46]	41	25	With aphasia	16	Picture	20
11	Brownell et al. (1990)‐1	17	M	CM		0.35 [−0.33, 1.03]	34	19	With aphasia	15	Written	16
12	Bryan (1988)‐2	18	M	CM		0.88 [0.35, 1.41]	60	30	With aphasia	30	Picture	10
13	Bryan (1988)‐3	18	M	CM		0.66 [0.14, 1.18]	60	30	With aphasia	30	Written	10
14	Cardillo et al. (2018)‐1	22	M	CM	Nominal‐Entity	−0.43 [−1.06, 0.19]	40	20	Without aphasia	20	Written	20
15	Cardillo et al. (2018)‐2	22	M	CM	Nominal‐Event	−0.56 [−1.19, 0.08]	40	20	Without aphasia	20	Written	20
16	Cardillo et al. (2018)‐3	22	M	CM	Predicate	−0.51 [−1.14, 0.12]	40	20	Without aphasia	20	Written	20
17	Giora et al. (2000)‐2	21	M	CM		−1.09 [−1.77, −0.41]	40	16	With aphasia	24	Verbal	4
18	Zaidel et al. (2002)‐3	21	M	CM		−0.33 [−1.03, 0.37]	35	12	With aphasia	23	Picture	?
19	Zaidel et al. (2002)‐4	21	M	CM		−0.95 [−1.69, −0.22]	35	12	With aphasia	23	Verbal	4
20	Winner and Gardner (1977)	16	M	CM		0.84 [0.28, 1.4]	57	35	With aphasia	22	Picture	18
21	Brundage (1993)‐2	21	L	Idiom	unfamiliar	−0.31 [−1.2, 0.57]	20	10	With aphasia	10	Verbal	12
22	Bryan (1988)‐1	18	L	Humor		0.39 [−0.12, 0.9]	60	30	With aphasia	30	Written	10
23	Giora et al. (2000)‐1	21	L	Irony		0.14 [−0.49, 0.77]	39	21	With aphasia	18	Judgement	5
24	IITAKA et al. (2003)‐3	20	L	Idiom	unfamiliar	0.14 [−0.62, 0.89]	36	27	With aphasia	9	Written	10
25	Shamay‐Tsoory et al. (2005)‐1	18	L	Irony		0.64 [−0.53, 1.8]	12	6	Without aphasia	6	Judgement	8
26	Shamay‐Tsoory et al. (2005)‐2	18	L	Irony		0.41 [−0.59, 1.41]	16	9	Without aphasia	7	Judgement	8
27	Zaidel et al. (2002)‐1	21	L	Humor		0.31 [−0.39, 1.02]	35	12	With aphasia	23	Picture	?
28	Zaidel et al. (2002)‐2	21	L	Humor		0.1 [−0.6, 0.8]	35	12	With aphasia	23	Written	?
29	Aydin et al. (2017)‐5	20	NF			−0.13 [−0.99, 0.73]	21	11	With aphasia	10	Written	20
30	Brownell et al. (1990)‐2	17	NF			−0.43 [−1.11, 0.26]	34	19	With aphasia	15	Written	16
31	Bryan (1988)‐4	18	NF			0.43 [−0.09, 0.94]	60	30	With aphasia	30	Picture	20
32	Cardillo et al. (2018)‐4	22	NF		Nominal‐Entity	−0.49 [−1.12, 0.14]	40	20	Without aphasia	20	Written	20
33	Cardillo et al. (2018)‐5	22	NF		Nominal‐Event	−0.31 [−0.93, 0.31]	40	20	Without aphasia	20	Written	20
34	Cardillo et al. (2018)‐6	22	NF		Predicate	−0.43 [−1.06, 0.2]	40	20	Without aphasia	20	Written	20
35	England (1986)‐2	20	NF			−0.37 [−1.08, 0.34]	31	15	With aphasia	16	Verbal	40
36	Kempler et al. (1999)‐2	22	NF			−0.85 [−1.5, −0.19]	41	25	With aphasia	16	Picture	20

Abbreviations: QA = quality assessment score (based on Kmet et al. 2004, 11‐item scale, maximum score = 22); Conventionality: H = high, M = medium, L = low, NF = non‐figurative/literal baseline; FLType = figurative language type; CM = conventional metaphor; FLSubType = figurative language subtype where applicable; LBD = left‐hemisphere damaged patients; RBD = right‐hemisphere damaged patients; Task types: Picture = multiple picture selection, Written = multiple sentence or word selection, Verbal = verbal meaning interpretation, Judgment = yes‐no question; n.Trial = number of trials per condition.

#### 3.1.1 Demographic and Clinical Information

As summarized in Table 3, age was matched or comparable between LBD and RBD groups in the majority of studies; only Winner and Gardner (1977) did not report age. Education was matched or comparable in most studies that reported this variable, though four studies did not report education. Lesion size, where reported, did not differ significantly between groups. Cognitive status was reported in a subset of studies using various instruments (e.g., MMSE, PBAC, RAPM, and WAIS‐R), but the heterogeneity of measures precluded systematic analysis. None of the included studies reported whether patients received cognitive or language treatment during the study period. Comorbid neurological conditions were explicitly excluded in most studies. These observations suggest that these variables are unlikely to have substantially confounded the direct LBD‐RBD comparison. However, time of assessment, aphasia severity, and lesion etiology were inconsistently reported and could not be systematically entered as moderators.

**TABLE 3:** Demographic and clinical information of studies included in the direct comparison between LBD and RBD patients.

StudyID	Age	Education	Time of assessment	Lesion size	Lesion etiology	Cognitive status	Comorbid neurological conditions
Aydin et al. (2017)	Matched (*p* = 0.634: 63.27 ± 11.14 vs. 65.3 ± 7.48)	Reported (literate to university vs. primary school to doctoral)	Matched (*p* = 0.77: 14.55 ± 4.59 vs. 15.10 ± 3.90)	Not reported	Stroke	Not reported	Excluded
Cardillo et al. (2018)	Matched (*p* > 0.05: 60.2 ± 11.9 vs. 62.8 ± 11.4)	Matched (*p* > 0.05: 14.3 ± 2.3 vs. 14.4 ± 2.6)	Matched (*p* > 0.05: 139.25 ± 42.22 vs. 168.60 ± 93.38)	Matched (*p* > 0.05: 37491.05 ± 45499.93 vs. 41921.40 ± 42356.68)	Stroke, hemorrhage, aneurysm, tumor resection	Matched	Excluded
Zaidel et al. (2002)	Controlled as covariate (57.7 ± 13.5 vs. 58.8 ± 9.8)	Reported (11.2 ± 3.8 vs. 10.7 ± 4.9)	Reported (11.9 ± 3.8 vs. 10.9 ± 3.8 weeks)	Controlled as covariate	Stroke	Described only (no formal group comparison)	Excluded
Giora et al. (2000)	Controlled as covariate (57.7 ± 13.5 vs. 58.8 ± 9.9)	Reported (11.2 ± 3.8 vs. 10.7 ± 4.9)	Reported (11.9 ± 3.8 vs. 10.9 ± 3.8 weeks)	Controlled as covariate	Stroke	Described only (no formal group comparison)	Excluded
Kempler et al. (1999)	Reported (58 vs. 65)	Not reported	Not reported	Not reported	Stroke	Not reported	Excluded
Brundage (1993)	Reported (69 vs. 66)	Reported (11.8 vs. 11.0)	Reported (19.7 vs. 8.7)	Not reported	Stroke	Not reported	Excluded
Brownell et al. (1990)	Reported (59.8 vs. 61.5)	Reported (12.3 vs. 11.9)	Reported (more than 2 months)	Not reported	Stroke	Not reported	Not reported
Bryan (1988)	Matched (no significant difference was found as *p* = 0.01)	Matched (no significant difference was found as *p* = 0.01)	Reported (test at 0–6 weeks post‐onset and retest at 14–20 weeks post‐onset)	Not reported	Stroke	Normal	Excluded
England (1986)	Matched (60.8 vs. 59.4)	Matched (11.8 vs. 12.2)	Reported (17 vs. 9.5)	Not reported	Stroke	Not reported	Excluded
Winner and Gardner (1977)	Not reported	Not reported	Not reported	Not reported	Not explicitly reported (presumed stroke)	Not reported	Not reported
IITAKA et al. (2003)	Reported (59.0 vs. 64.7)	Reported (range only)	Not reported	Not reported	Stroke, traumatic brain injury (1 case)	Reported (WAIS‐R Picture Arrangement: significant lower for RBD)	Excluded
Murphy et al. (2013)	Matched (46.48 ± 11.26 vs. 45.61 ± 15.76)	Not reported	Reported (18.16 ± 26.05 vs. 22.77 ± 34.22)	Reported (lesion regions count: 3.11 ± 2.35 vs. 2.70 ± 1.66)	Stroke, tumor resection (mixed)	Reported (LBD impaired on Trails B & Stroop; RBD unimpaired vs. HC's; RAPM preserved in both	Excluded
Shamay‐Tsoory et al. (2005)	Matched (oral reported)	Matched (oral reported)	Reported (more than 6 months)	Matched (*p* = 0.854: 2.70 ± 2.39 vs. 2.87 ± 2.40)	Traumatic brain injury, tumor, stroke (mixed)	Normal	Excluded

*Note*: Zaidel et al. (2002) and Giora et al. (2000) drew from the same patient cohort; therefore, their demographic and clinical characteristics are identical.

#### 3.1.2 Overall Analysis and Conventionality Subgroups

We first conducted an overall analysis combining all figurative language types (high, medium, and low conventionality) to examine whether LBD and RBD patients differed in their figurative language comprehension performance. The pooled effect size across the 28 studies was not significant (Hedges’ *g* = −0.044, 95% CI [−0.331, 0.242], *p* = 0.763), indicating that, overall, LBD and RBD patients did not differ in their accuracy on figurative language comprehension tasks. However, significant heterogeneity was observed (*Q*(27) = 113.047, *p* < 0.001, *I* ^2^ = 78.1%), suggesting substantial variability in effect sizes across studies that warranted further investigation through subgroup analyses.

We next examined whether the pattern of hemispheric differences varied as a function of conventionality. Although the interaction between conventionality and lesion side did not reach statistical significance (as indicated by overlapping confidence intervals across subgroups), subgroup analyses revealed interesting trends (see Figure 2 for individual study effect sizes and Figure 3 for pooled effects by group). At the high‐conventionality level (*k* = 10), the pooled effect size was not significant (Hedges’ *g* = −0.242, 95% CI [−0.935, 0.451], *p* = 0.493), with high heterogeneity (*Q*(9) = 56.624, *p* < 0.001, *I* ^2^ = 86.7%). At the medium‐conventionality level (*k* = 10), the effect was also not significant (Hedges’ *g* = −0.092, 95% CI [−0.552, 0.369], *p* = 0.697), with substantial heterogeneity (*Q*(9) = 50.877, *p* < 0.001, *I* ^2^ = 81.8%). At the low‐conventionality level (*k* = 8), a trend toward better performance of LBD patients compared to RBD patients was observed (Hedges’ *g* = 0.225, 95% CI [−0.032, 0.481], *p* = 0.086), with no heterogeneity (*Q*(7) = 2.722, *p* = 0.910, *I* ^2^ = 0%). For the non‐figurative/literal baseline condition (*k* = 8), a significant effect emerged, with worse performance of LBD patients compared to RBD patients (Hedges’ *g* = −0.302, 95% CI [−0.600, −0.004], *p* = 0.047), with moderate heterogeneity (*Q*(7) = 11.226, *p* = 0.129, *I* ^2^ = 40.2%).

**FIGURE 2:**
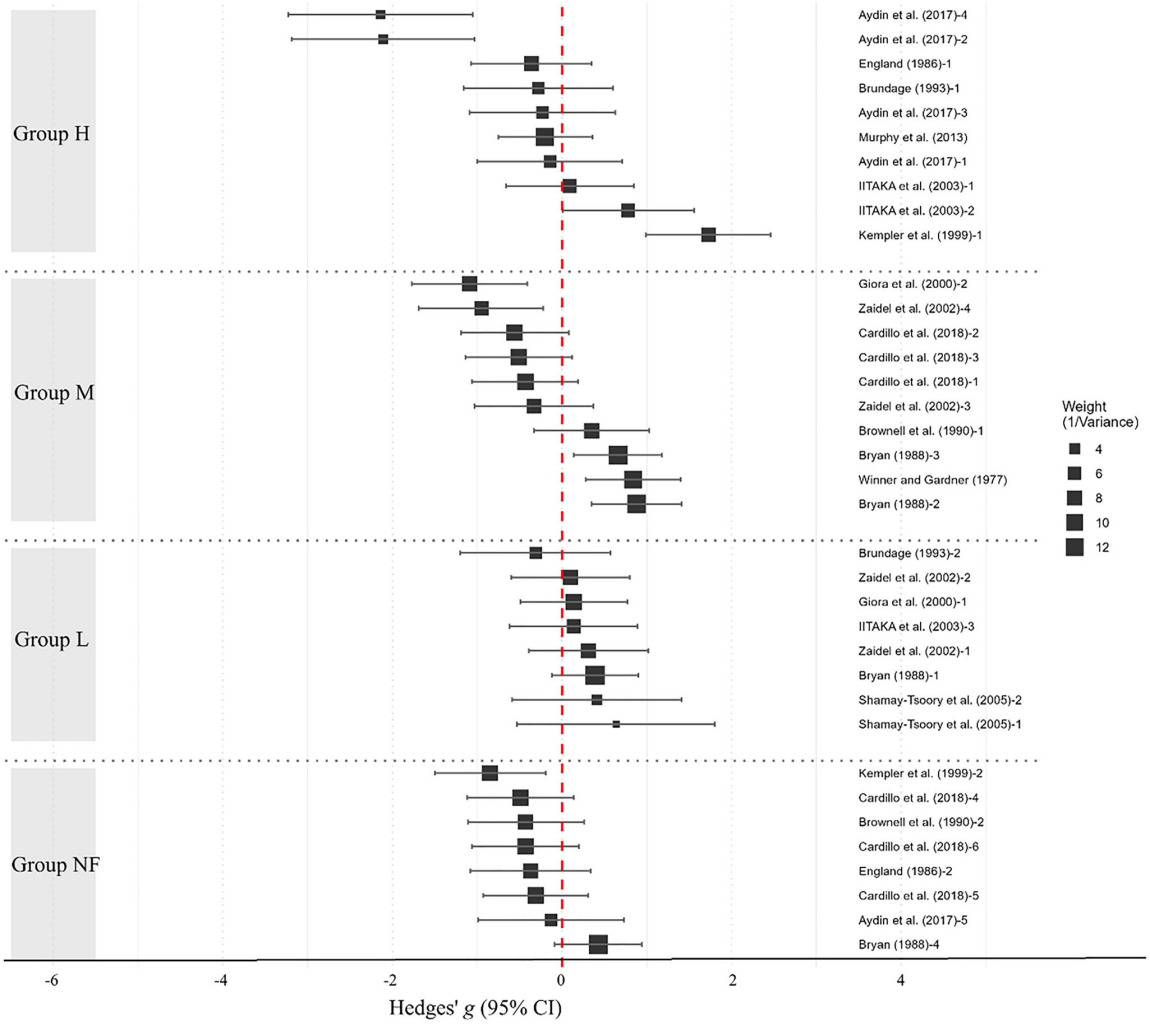
Forest plot of individual study effect sizes by conventionality group (random‐effects model). (H: high conventionality, *k* = 10; M: medium conventionality, *k* = 10; L: low conventionality, *k* = 8; NF: non‐figurative, *k* = 8. Negative values indicate poorer performance in the LBD group relative to RBD.)

**FIGURE 3:**
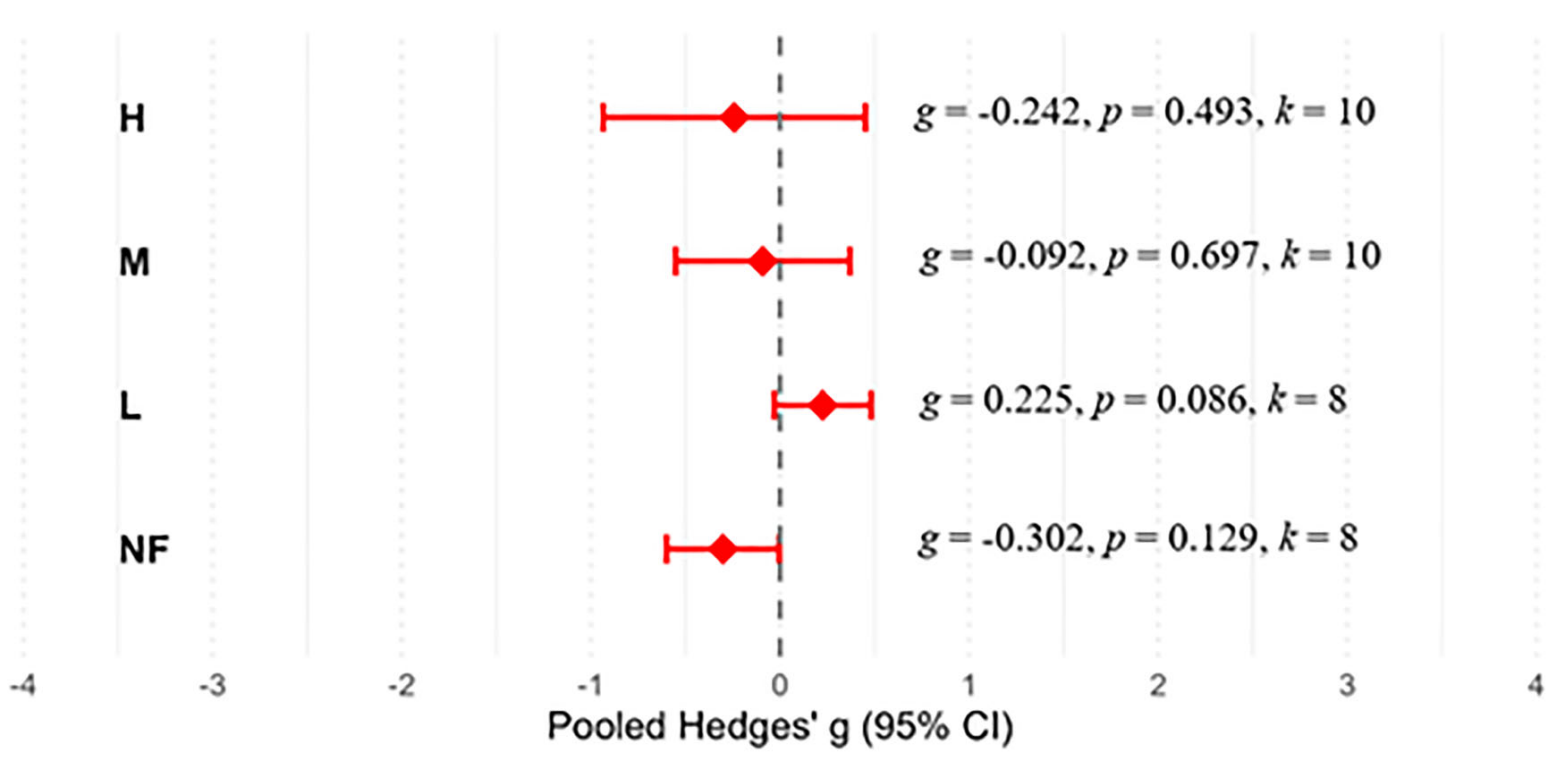
Forest plot of pooled effect sizes by conventionality group (random‐effects model). (H = high conventionality; M = medium conventionality; L = low conventionality; NF = non‐figurative/literal language.)

#### 3.1.3 Sensitivity Analysis and Publication Bias

To assess the robustness of the pooled effect sizes and to identify potentially influential studies, influence analysis was conducted using the leave‐one‐out method (Viechtbauer and Cheung 2010). For the overall analysis combining all figurative language types (*k* = 28), the leave‐one‐out estimates ranged from −0.105 to 0.019, all remaining within the 95% confidence interval of the original pooled estimate (−0.333 to 0.242). No single study was identified as a potential outlier or as having disproportionate influence on the overall effect size. Subgroup analyses by conventionality level similarly revealed no influential outliers: for high‐conventionality (*k* = 10, original *g* = −0.244), medium‐conventionality (*k* = 10, original *g* = −0.092), low‐conventionality (*k* = 8, original *g* = 0.224), and non‐figurative baseline (*k* = 8, original *g* = −0.302) items, all leave‐one‐out estimates remained within the confidence intervals of the respective original estimates. These findings confirm the robustness of the meta‐analytic results and indicate that no single study unduly influenced the observed effect sizes.

Publication bias was assessed using funnel plots and Egger's regression tests. For the overall analysis combining all figurative language types, Egger's test indicated significant asymmetry (*z* = −2.045, *p* = 0.041). The trim‐and‐fill method imputed zero studies, and the adjusted effect size remained unchanged (Hedges’ *g* = −0.044, 95% CI [−0.331, 0.242]), suggesting that the overall finding was robust to publication bias.

For the subgroup analyses, Egger's tests revealed significant asymmetry only for the medium‐conventionality group (*z* = −4.035, *p* = 0.004; see Figure 4 for the funnel plot). However, trim‐and‐fill analysis did not impute any missing studies (estimated missing studies on the right side = 0, *SE* = 2.085), and the adjusted effect size (Hedges’ *g* = −0.092, 95% CI [−0.553, 0.369], *p* = 0.696) was identical to the original estimate. This suggests that the observed asymmetry in the medium‐conventionality group is likely attributable to between‐study heterogeneity (*I* ^2^ = 81.8%) rather than publication bias. No significant asymmetry was detected for the high‐ (*p* = 0.147), low‐ (*p* = 0.862), or non‐figurative (*p* = 0.198) groups.

**FIGURE 4:**
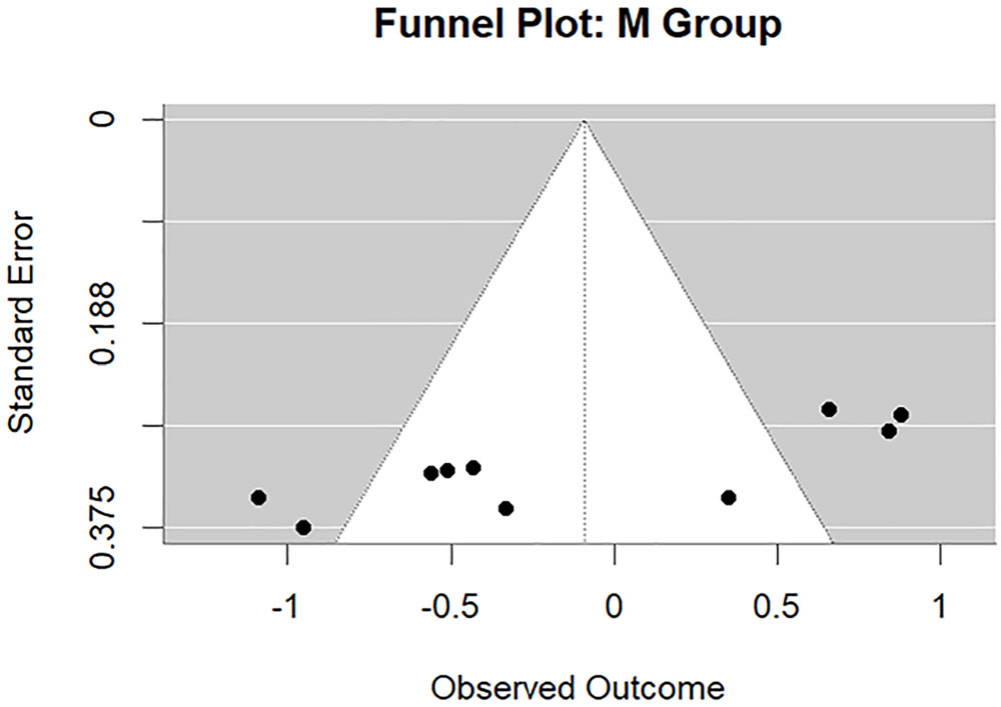
Funnel plot for the medium conventionality group.

#### 3.1.4 Moderator Analyses

To examine whether the overall LBD‐RBD difference in figurative language comprehension was moderated by study‐level factors, we conducted meta‐regressions with LBD subtype (aphasic vs. non‐aphasic), sample size, task type, and number of trials as predictors. These analyses were performed at the overall level (i.e., collapsing across conventionality levels) rather than stratified by conventionality subgroups, as the limited number of studies available for subgroup analyses precluded reliable stratified estimates (Cuijpers et al. 2021). Only figurative language studies were included in these analyses (*k* = 28, excluding NF/literal baseline conditions).

As shown in Figure 5, LBD subtype (aphasic vs. non‐aphasic) did not significantly moderate the overall effect (*QM*(1) = 0.816, *p* = 0.366), suggesting that the presence of aphasia did not systematically alter the pattern of hemispheric differences in figurative language comprehension.

**FIGURE 5:**
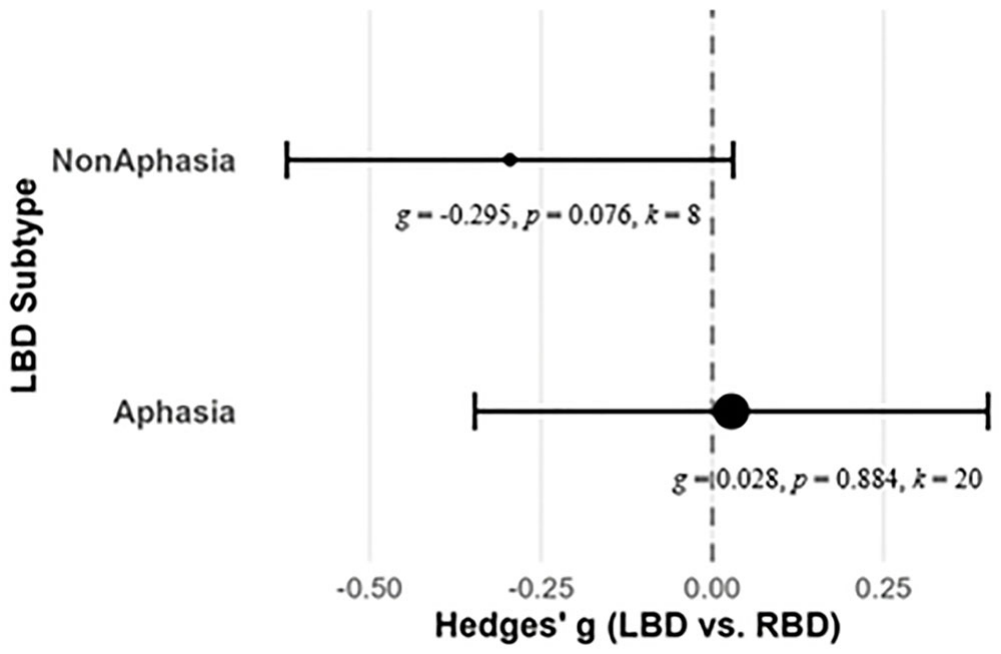
Forest plot of individual study effect sizes by LBD subtype (random‐effects model). (*QM*(1) = 0.816, *p* = 0.366; LBD = left‐brain‐damage patients; RBD = right‐brain‐damage patients).

As shown in Figure 6, sample size significantly moderated the overall effect (slope = 0.024, *p* = 0.017), with larger samples associated with more positive effect sizes (i.e., reduced LBD disadvantage or enhanced LBD advantage relative to RBD).

**FIGURE 6:**
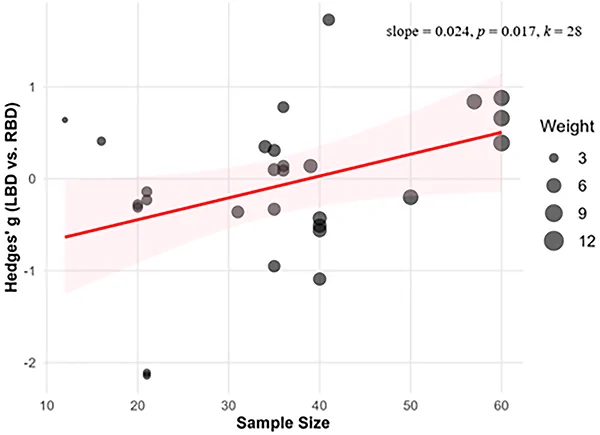
Scatter plot of effect sizes as a function of sample size (meta‐regression). (LBD = left‐brain‐damage patients; RBD = right‐brain‐damage patients).

As shown in Figure 7, task type significantly moderated the overall effect (*QM*(3) = 8.93, *p* = 0.030). Post hoc subgroup analyses revealed that this moderating effect was primarily driven by verbal meaning explanation tasks, which showed a significant LBD disadvantage (Hedges’ *g* = −0.638, 95% CI [−1.025, −0.251], *p* = 0.001, *k* = 8). In contrast, no significant LBD‐RBD differences were observed in multiple picture selection tasks (Hedges’ *g* = 0.215, 95% CI [−0.640, 1.070], *p* = 0.622, *k* = 7), multiple sentence/word selection tasks (Hedges’ *g* = 0.101, 95% CI [−0.201, 0.403], *p* = 0.513, *k* = 10), or yes–no question tasks (Hedges’ *g* = 0.290, 95% CI [−0.195, 0.775], *p* = 0.241, *k* = 3). Substantial heterogeneity was observed in the multiple picture selection tasks (*I* ^2^ = 90.4%), whereas the verbal meaning explanation (*I* ^2^ = 49.9%), multiple sentence/word selection (*I* ^2^ = 54.0%), and yes–no question (*I* ^2^ = 0%) tasks showed moderate to low heterogeneity.

**FIGURE 7:**
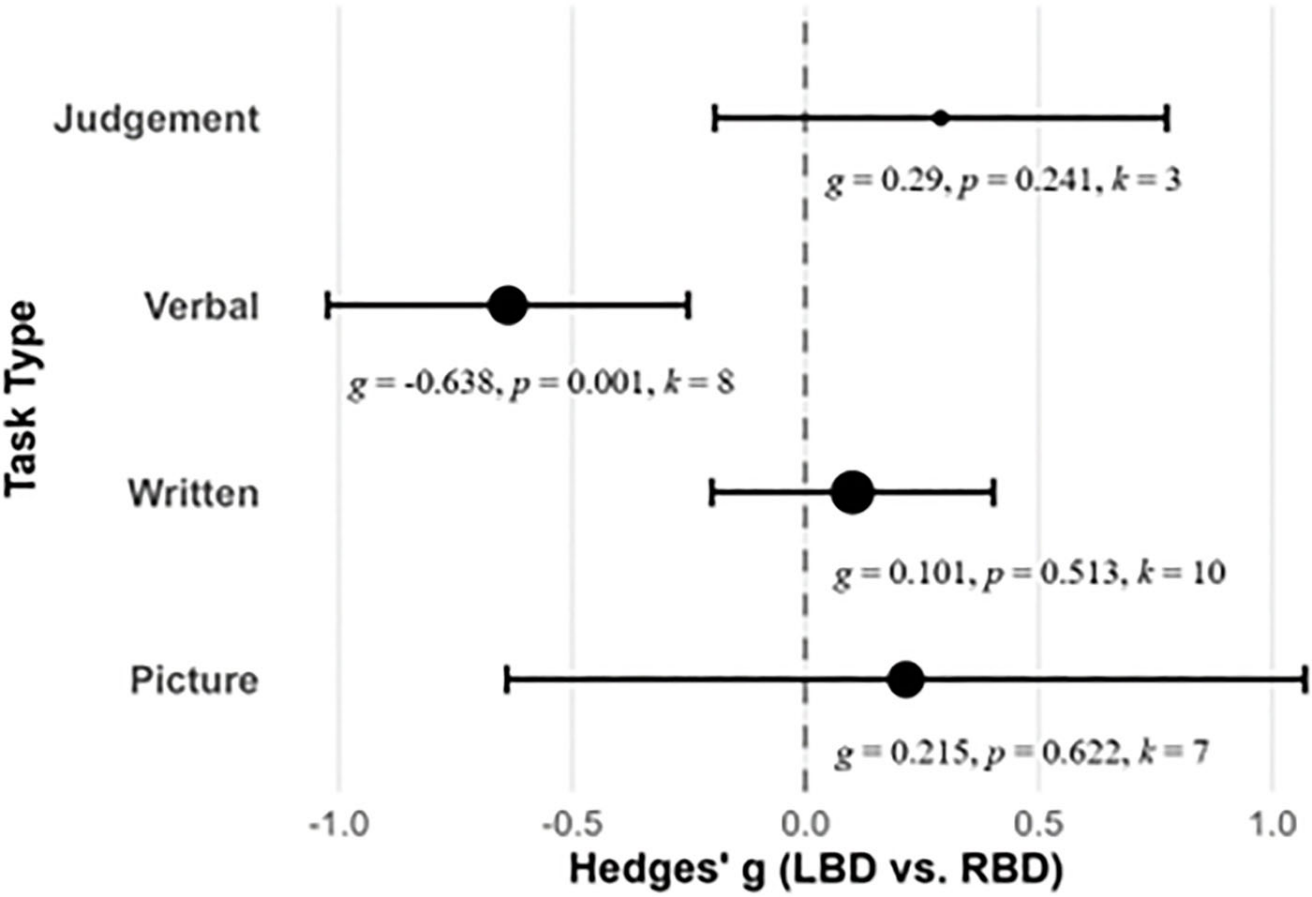
Forest plot of individual study effect sizes by task type (random‐effects model). (*QM*(3) = 8.93, *p* = 0.030; Judgement = yes–no question; Verbal = verbal meaning explanation; Written = multiple sentence/word selection; Picture = multiple picture selection; LBD = left‐brain‐damage patients; RBD = right‐brain‐damage patients).

As shown in Figure 8, number of trials did not significantly moderate the overall effect (slope = −0.032, *p* = 0.093), indicating that task length did not systematically influence hemispheric differences within the range of trial numbers represented in the included studies.

**FIGURE 8:**
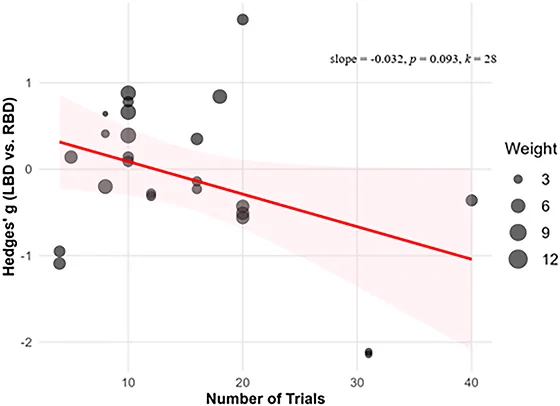
Scatter plot of effect sizes as a function of number of trials (meta‐regression).

### 3.2 Indirect Comparisons: LBD Versus Healthy Controls and RBD Versus Healthy Controls

For the indirect comparisons, 70 studies contributed to the LBD versus HC analysis, including 1120 LBD patients and 1342 healthy controls (see Table 4). The RBD versus HC analysis included 68 studies, with 1135 RBD patients and 1291 healthy controls (see Table 5).^3^ Multi‐level random‐effects models were used to compute pooled effect sizes for each conventionality level separately for the two patient groups, following the same analytic approach as in the direct comparisons. Figure 9 provides a visual comparison of the impairment patterns across groups and conventionality levels.

**TABLE 4:** Summary of studies included in the comparison between LBD patients and healthy controls.

No	StudyID	QA	Conventionality	FLType	FLSubType	Hedges’ *g* [95% CI]	Sample Size	n.LBD	LBDSubType	n.HC	Task	n.Trial
1	Aydin et al. (2017)‐1	20	H	Idiom	Transparent	−1.53 [−2.55, −0.51]	22	11	With aphasia	11	Picture	16
2	Aydin et al. (2017)‐2	20	H	Idiom	Opaque	−5.96 [−8.11, −3.8]	22	11	With aphasia	11	Picture	31
3	Aydin et al. (2017)‐3	20	H	Idiom	Transparent	−1.63 [−2.67, −0.59]	22	11	With aphasia	11	Verbal	16
4	Aydin et al. (2017)‐4	20	H	Idiom	Opaque	−5.31 [−7.28, −3.35]	22	11	With aphasia	11	Verbal	31
5	Borelli et al. (2022)‐1	19	H	Dead metaphor	Action metaphors	−0.59 [−1.29, 0.12]	37	14	Without aphasia	23	Picture	12
6	Borelli et al. (2022)‐2	19	H	Dead metaphor	Non‐action metaphors	−0.2 [−0.89, 0.49]	37	14	Without aphasia	23	Picture	6
7	Brundage (1993)‐1	21	H	Idiom	Familiar	−1.2 [−2.06, −0.34]	30	10	With aphasia	20	Verbal	12
8	Cacciari et al. (2006)	21	H	Idiom		−2.04 [−2.97, −1.11]	30	15	With aphasia	15	Written	23
9	Chapman et al. (1997)‐1	22	H	Idiom	Familiar	−1.45 [−2.51, −0.38]	20	10	With aphasia	10	Verbal	6
10	Chapman et al. (1997)‐2	22	H	Idiom	Familiar	−0.43 [−1.38, 0.52]	20	10	With aphasia	10	Written	2
11	England (1986)‐1	20	H	Proverb		−1.09 [−1.91, −0.27]	29	15	With aphasia	14	Verbal	40
12	IITAKA et al. (2003)‐1	20	H	Idiom	Familiar	−2.28 [−3.08, −1.49]	42	27	With aphasia	15	Written	10
13	IITAKA et al. (2003)‐2	20	H	Idiom	Familiar	−1.6 [−2.32, −0.88]	42	27	With aphasia	15	Written	10
14	Milburn et al. (2018)‐1	21	H	Idiom		−0.8 [−1.44, −0.16]	47	16	With aphasia	31	Written	5
15	Milburn et al. (2018)‐2	21	H	Idiom		−1.04 [−1.7, −0.38]	47	16	With aphasia	31	Written	5
16	Morawski (2009)‐1	10	H	Idiom		−3.07 [−6.84, 0.7]	6	3	With aphasia	3	Written	30
17	Morawski (2009)‐2	10	H	Idiom		−3.39 [−7.42, 0.64]	6	3	With aphasia	3	Written	30
18	Murphy et al. (2013)	22	H	Proverb		−0.67 [−1.14, −0.19]	79	27	Without aphasia	52	Verbal	8
19	Papagno and Caporali (2007)‐1	22	H	Idiom	Unambiguous	−1.65 [−2.52, −0.77]	30	15	With aphasia	15	Picture	40
20	Papagno and Caporali (2007)‐2	22	H	Idiom	Unambiguous	−1.45 [−2.3, −0.61]	30	15	With aphasia	15	Written	40
21	Papagno and Caporali (2007)‐3	22	H	Idiom	Unambiguous	−1.77 [−2.66, −0.87]	30	15	With aphasia	15	Verbal	40
22	Papagno and Caporali (2007)‐4	22	H	Idiom	Ambiguous	−1.37 [−2.2, −0.53]	30	15	With aphasia	15	Written	15
23	Papagno and Genoni (2004)	21	H	Idiom	Opaque idioms, without context	−1.41 [−2.42, −0.41]	22	11	With aphasia	11	Picture	40
24	Papagno et al. (2006)‐1	22	H	Idiom		−2.52 [−3.67, −1.36]	24	12	With aphasia	12	Picture	40
25	Papagno et al. (2006)‐2	22	H	Idiom		−2.18 [−3.27, −1.09]	24	12	With aphasia	12	Verbal	40
26	Tan et al. (2024)‐1	19	H	Idiom		−4.5 [−6.23, −2.77]	22	11	With aphasia	11	Picture	16
27	Tan et al. (2024)‐2	19	H	Idiom		−1.16 [−2.12, −0.19]	22	11	With aphasia	11	Written	16
28	Ulatowska et al. (2000)‐1	20	H	Proverb		−0.65 [−1.27, −0.04]	46	23	With aphasia	23	Written	20
29	Ulatowska et al. (2000)‐2	20	H	Proverb		−0.22 [−0.82, 0.37]	46	23	With aphasia	23	Written	6
30	Ulatowska et al. (2000)‐3	20	H	Proverb		−1.01 [−1.64, −0.37]	46	23	With aphasia	23	Written	10
31	Ulatowska et al. (2000)‐4	20	H	Proverb	Core proverbs	−0.65 [−1.26, −0.04]	46	23	With aphasia	23	Verbal	10
32	Ulatowska et al. (2000)‐5	20	H	Proverb	Animal proverbs	−0.27 [−0.87, 0.32]	46	23	With aphasia	23	Verbal	10
33	Ulatowska et al. (2000)‐6	20	H	Proverb	Weather proverbs	−0.16 [−0.75, 0.44]	46	23	With aphasia	23	Verbal	10
34	Ulatowska et al. (2001)‐1	20	H	Proverb	Non‐ambiguous idiom	−1.13 [−1.64, −0.62]	72	36	With aphasia	36	Verbal	5
35	Ulatowska et al. (2001)‐2	20	H	Proverb	Non‐ambiguous idiom	−0.26 [−0.73, 0.22]	72	36	With aphasia	36	Written	5
36	Borelli et al. (2022)‐5	19	M	CM	Action metaphors	−1.1 [−1.84, −0.36]	37	14	Without aphasia	23	Picture	12
37	Borelli et al. (2022)‐6	19	M	CM	Non‐action metaphors	−0.71 [−1.42, 0]	37	14	Without aphasia	23	Picture	6
38	Bryan (1988)‐2	18	M	CM		−1.19 [−1.75, −0.63]	60	30	With aphasia	30	Picture	10
39	Bryan (1988)‐3	18	M	CM		−0.99 [−1.54, −0.45]	60	30	With aphasia	30	Written	10
40	Cardillo et al. (2018)‐1	22	M	CM	Nominal entity	−0.88 [−1.55, −0.21]	40	20	Without aphasia	20	Written	20
41	Cardillo et al. (2018)‐2	22	M	CM	Nominal event	−0.62 [−1.27, 0.04]	40	20	Without aphasia	20	Written	20
42	Cardillo et al. (2018)‐3	22	M	CM	Predicate	−0.45 [−1.1, 0.2]	40	20	Without aphasia	20	Written	20
43	Henderson and Wright (2016)‐1	18	M	CM	Dominant metaphor	−2.22 [−3.64, −0.79]	16	6	With aphasia	10	Judgement	14
44	Henderson and Wright (2016)‐2	18	M	CM	Subordinate metaphor	−1.67 [−2.97, −0.37]	16	6	With aphasia	10	Judgement	14
45	Zaidel et al. (2002)‐3	21	M	CM		−2.76 [−3.79, −1.73]	33	12	Without aphasia	21	Picture	?
46	Zaidel et al. (2002)‐4	21	M	CM		−1.66 [−2.52, −0.81]	33	12	Without aphasia	21	Verbal	4
47	Borelli et al. (2022)‐3	19	L	Novel metaphor	Action metaphors	−0.89 [−1.61, −0.17]	37	14	Without aphasia	23	Picture	12
48	Borelli et al. (2022)‐4	19	L	Novel metaphor	Non‐action metaphors	−0.45 [−1.15, 0.25]	37	14	Without aphasia	23	Picture	6
49	Brundage (1993)‐2	21	L	Idiom	Unfamiliar	−1.09 [−1.94, −0.24]	30	10	With aphasia	20	Verbal	12
50	Bryan (1988)‐1	18	L	humor		−2.17 [−2.82, −1.51]	60	30	With aphasia	30	Written	10
51	Chapman et al. (1997)‐3	22	L	Idiom	Unfamiliar	−1.64 [−2.74, −0.54]	20	10	With aphasia	10	Verbal	6
52	Chapman et al. (1997)‐4	22	L	Idiom	Unfamiliar	−0.51 [−1.47, 0.45]	20	10	With aphasia	10	Written	2
53	IITAKA et al. (2003)‐3	20	L	Idiom	Unfamiliar	−2.27 [−3.07, −1.47]	42	27	With aphasia	15	Written	10
54	Milburn et al. (2018)‐3	21	L	Idioms	Unfamiliar	−0.79 [−1.43, −0.15]	47	16	With aphasia	31	Written	5
55	Milburn et al. (2018)‐4	21	L	Idioms	Unfamiliar	−0.63 [−1.26, 0.01]	47	16	With aphasia	31	Written	5
56	Shamay‐Tsoory et al. (2005)‐1	18	L	Irony		−0.84 [−1.8, 0.12]	23	6		17	Judgement	8
57	Shamay‐Tsoory et al. (2005)‐2	18	L	Irony		0 [−0.81, 0.81]	26	9		17	Judgement	8
58	Zaidel et al. (2002)‐1	21	L	Humor		−0.41 [−1.16, 0.33]	33	12	Without aphasia	21	Picture	?
59	Zaidel et al. (2002)‐2	21	L	Humor		−2.5 [−3.48, −1.52]	33	12	Without aphasia	21	Written	?
60	Aydin et al. (2017)‐5	20	NF			−2.03 [−3.14, −0.92]	22	11	With aphasia	11	Written	20
61	Bryan (1988)‐4	18	NF			−0.93 [−1.47, −0.38]	60	30	With aphasia	30	Picture	20
62	Cardillo et al. (2018)‐4	22	NF			−0.41 [−1.06, 0.24]	40	20	Without aphasia	20	Written	20
63	Cardillo et al. (2018)‐5	22	NF			−0.52 [−1.17, 0.14]	40	20	Without aphasia	20	Written	20
64	Cardillo et al. (2018)‐6	22	NF			−0.32 [−0.97, 0.32]	40	20	Without aphasia	20	Written	20
65	England (1986)‐2	20	NF			−1.13 [−1.96, −0.31]	29	15	With aphasia	14	Verbal	40
66	Henderson and Wright (2016)‐3	18	NF			−0.91 [−2.07, 0.26]	16	6	With aphasia	10	Judgement	14
67	Henderson and Wright (2016)‐4	18	NF			−1.18 [−2.39, 0.02]	16	6	With aphasia	10	Judgement	14
68	Milburn et al. (2018)‐5	21	NF			−0.26 [−0.88, 0.37]	47	16	With aphasia	31	Written	5
69	Milburn et al. (2018)‐6	21	NF			−0.64 [−1.27, 0]	47	16	With aphasia	31	Written	5
70	Papagno et al. (2006)‐5	22	NF			−1.88 [−2.91, −0.85]	24	12	With aphasia	12	Picture	40

Abbreviations: QA = quality assessment score (based on Kmet et al. 2004, 11‐item scale, maximum score = 22); Conventionality: H = high, M = medium, L = low, NF = non‐figurative/literal baseline; FLType = figurative language type; CM = conventional metaphor; FLSubType = figurative language subtype where applicable; LBD = left‐hemisphere damaged patients; HC = healthy controls; Task types: Picture = multiple picture selection, Written = multiple sentence or word selection, Verbal = verbal meaning interpretation, Judgment = yes‐no question; n.Trial = number of trials per condition.

**TABLE 5:** Summary of studies included in the comparison between RBD patients and healthy controls.

No	StudyID	QA	Conventionality	FLType	FLSubType	Hedges’ *g* [95% CI]	Sample Size	n.RBD	n.HC	Task	n.Trial
1	Aydin et al. (2017)‐1	20	H	Idiom	Transparent	−3.29 [−4.6, −1.98]	21	10	11	Picture	16
2	Aydin et al. (2017)‐2	20	H	Idiom	Opaque	−2.67 [−3.84, −1.49]	21	10	11	Picture	31
3	Aydin et al. (2017)‐3	20	H	Idiom	Transparent	−2.32 [−3.43, −1.22]	21	10	11	Verbal	16
4	Aydin et al. (2017)‐4	20	H	Idiom	Opaque	−2.87 [−4.08, −1.65]	21	10	11	Verbal	31
5	Brundage (1993)‐1	21	H	Idiom	Familiar	−0.94 [−1.73, −0.14]	30	10	20	Verbal	12
6	Chakrabarty et al. (2023)‐1	19	H	Idiom		−4.74 [−6.14, −3.34]	30	15	15	Picture & Verbal	11
7	England (1986)‐1	20	H	Proverb		−0.91 [−1.66, −0.16]	30	16	14	Verbal	40
8	Fedanick (2001)‐1	20	H	Idiom		−2.38 [−3.52, −1.23]	20	10	10	Verbal	10
9	Fedanick (2001)‐2	20	H	Idiom		−1.62 [−2.63, −0.61]	20	10	10	Verbal	10
10	Hier and Kaplan (1980)‐1	21	H	Proverb		−0.89 [−1.51, −0.27]	50	34	16	Verbal	12
11	IITAKA et al. (2003)‐1	20	H	Idiom	Familiar	−1.94 [−2.93, −0.95]	24	9	15	Written	10
12	IITAKA et al. (2003)‐2	20	H	Idiom	Familiar	−1.9 [−2.88, −0.91]	24	9	15	Written	10
13	Murphy et al. (2013)	22	H	Proverb		−0.48 [−0.98, 0.02]	75	23	52	Verbal	8
14	Papagno et al. (2006)‐3	22	H	Idiom		−1.02 [−1.78, −0.26]	30	15	15	Picture	40
15	Papagno et al. (2006)‐4	22	H	Idiom		−0.46 [−1.19, 0.26]	30	15	15	Verbal	40
16	Priestap‐Clausen (2000)‐1	20	H	Idiom		−3.56 [−5.32, −1.79]	13	8	5	Verbal	4
17	Bricker (1999)‐2	21	M	Pun	Conventional	−1.58 [−2.64, −0.52]	18	9	9	Judgement	10
18	Bryan (1988)‐2	18	M	CM		−1.98 [−2.6, −1.36]	60	30	30	Picture	10
19	Bryan (1988)‐3	18	M	CM		−1.53 [−2.11, −0.96]	60	30	30	Written	10
20	Cardillo et al. (2018)‐1	22	M	CM	Nominal entity	−0.32 [−0.94, 0.3]	40	20	20	Written	20
21	Cardillo et al. (2018)‐2	22	M	CM	Nominal event	−0.07 [−0.69, 0.55]	40	20	20	Written	20
22	Cardillo et al. (2018)‐3	22	M	CM	Predicate	0.08 [−0.54, 0.7]	40	20	20	Written	20
23	Lee et al. (2009)‐1	19	M	CM		−4.79 [−6.37, −3.22]	24	12	12	Picture	20
24	Lee et al. (2009)‐2	19	M	CM	Without context	−2 [−2.99, −1.02]	24	12	12	Written	20
25	Lee et al. (2009)‐3	19	M	CM	With context	−1.3 [−2.18, −0.42]	24	12	12	Written	20
26	Mackenzie and Brady (2004)	19	M	CM	With context	−0.75 [−1.56, 0.06]	29	9	20	Picture	10
27	Mackenzie et al. (1997)‐2	21	M	CM		−0.78 [−1.49, −0.07]	34	14	20	Picture	10
28	Mackenzie et al. (1999)‐1	20	M	CM		−1.12 [−1.6, −0.64]	76	36	40	Picture	10
29	Mackenzie et al. (1999)‐2	20	M	CM		−0.53 [−1.26, 0.19]	32	12	20	Picture	10
30	Shin and Choi (2017)‐2	20	M	CM		−2.15 [−3.15, −1.14]	24	12	12	Written	15
31	Zaidel et al. (2002)‐3	21	M	CM		−1.53 [−2.2, −0.86]	44	23	21	Picture	?
32	Zaidel et al. (2002)‐4	21	M	CM		−0.9 [−1.52, −0.28]	44	23	21	Verbal	4
33	Bricker (1999)‐1	21	L	Pun	Novel	−0.75 [−1.7, 0.21]	18	9	9	Judgement	10
34	Brundage (1993)‐2	21	L	Idiom	Unfamiliar	−0.75 [−1.53, 0.04]	30	10	20	Verbal	12
35	Bryan (1988)‐1	18	L	Humor		−1.72 [−2.31, −1.13]	60	30	30	Written	10
36	Chakrabarty et al. (2023)‐2	19	L	Humor		−4.2 [−5.48, −2.92]	30	15	15	Verbal	4
37	Champagne‐Lavau et al. (2018)‐1	21	L	Irony		−0.99 [−1.64, −0.33]	40	20	20	Verbal	12
38	Champagne‐Lavau et al. (2018)‐2	21	L	Irony		−0.84 [−1.49, −0.19]	40	20	20	Verbal	12
39	Champagne‐Lavau et al. (2018)‐3	21	L	Irony		−0.28 [−0.91, 0.34]	40	20	20	Verbal	12
40	Cheang and Pell (2006)‐1	21	L	Joke		−0.7 [−1.61, 0.2]	20	10	10	Written	16
41	Frau et al. (2025)	22	L	Humor		−0.73 [−1.06, −0.39]	170	51	119	Written	7
42	IITAKA et al. (2003)‐3	20	L	Idiom	Unfamiliar	−2.24 [−3.29, −1.2]	24	9	15	Written	10
43	Kim and Choi (2009)‐1	19	L	Novel metaphor		−1.57 [−2.42, −0.72]	28	14	14	Written	20
44	Mackenzie et al. (1997)‐1	21	L	Humor		0.04 [−0.65, 0.72]	34	14	20	Written	10
45	Martin and McDonald (2006)‐1	21	L	Joke		−1.56 [−2.25, −0.87]	42	21	21	Judgement	3
46	Shamay‐Tsoory et al. (2005)‐1	18	L	Irony		−1.99 [−3.09, −0.9]	23	6	17	Judgement	8
47	Shamay‐Tsoory et al. (2005)‐2	18	L	Irony		−0.45 [−1.34, 0.44]	24	7	17	Judgement	8
48	Shin and Choi (2017)‐1	20	L	Novel metaphor		−2.65 [−3.74, −1.55]	24	12	12	Written	15
49	Winner et al. (1998)‐1	22	L	Joke		−0.26 [−0.97, 0.44]	33	13	20	Judgement	7
50	Zaidel et al. (2002)‐1	21	L	Humor		−0.72 [−1.34, −0.11]	44	23	21	Picture	?
51	Zaidel et al. (2002)‐2	21	L	Humor		−1.68 [−2.37, −0.99]	44	23	21	Written	?
52	Aydin et al. (2017)‐5	20	NF			−1.67 [−2.66, −0.67]	21	10	11	Written	20
53	Bricker (1999)‐3	21	NF			−1.46 [−2.5, −0.42]	18	9	9	Judgement	10
54	Bryan (1988)‐4	18	NF			−1.67 [−2.26, −1.08]	60	30	30	Picture	20
55	Cardillo et al. (2018)‐4	22	NF			0.08 [−0.54, 0.7]	40	20	20	Written	20
56	Cardillo et al. (2018)‐5	22	NF			−0.21 [−0.83, 0.42]	40	20	20	Written	20
57	Cardillo et al. (2018)‐6	22	NF			0.22 [−0.4, 0.84]	40	20	20	Written	20
58	Champagne‐Lavau et al. (2018)‐4	21	NF			−0.08 [−0.7, 0.54]	40	20	20	Judgement	12
59	Champagne‐Lavau et al. (2018)‐5	21	NF			−0.34 [−0.96, 0.29]	40	20	20	Judgement	12
60	Champagne‐Lavau et al. (2018)‐6	21	NF			0.29 [−0.33, 0.91]	40	20	20	Judgement	12
61	Cheang and Pell (2006)‐2	21	NF			−0.45 [−1.34, 0.44]	20	10	10	Written	16
62	England (1986)‐2	20	NF			−0.66 [−1.4, 0.07]	30	16	14	Verbal	40
63	Hier and Kaplan (1980)‐2	21	NF			−0.03 [−0.63, 0.56]	50	34	16	Verbal	40
64	Kim and Choi (2009)‐2	19	NF			−1.45 [−2.28, −0.62]	28	14	14	Written	20
65	Martin and McDonald (2006)‐2	21	NF			−0.37 [−0.98, 0.24]	42	21	21	Judgement	3
66	Papagno et al. (2006)‐6	22	NF			−0.63 [−1.37, 0.1]	30	15	15	Picture	40
67	Priestap‐Clausen (2000)‐2	20	NF			−0.83 [−1.99, 0.33]	13	8	5	Verbal	5
68	Winner et al. (1998)‐2	22	NF			−0.84 [−1.57, −0.11]	33	13	20	Judgement	8

Abbreviations: QA = quality assessment score (based on Kmet et al. 2004, 11‐item scale, maximum score = 22); Conventionality: H = high, M = medium, L = low, NF = non‐figurative/literal baseline; FLType = figurative language type; CM = conventional metaphor; FLSubType = figurative language subtype where applicable; RBD = right‐hemisphere damaged patients; HC = healthy controls; Task types: Picture = multiple picture selection, Written = multiple sentence or word selection, Verbal = verbal meaning interpretation, Judgment = yes‐no question; n.Trial = number of trials per condition.

**FIGURE 9:**
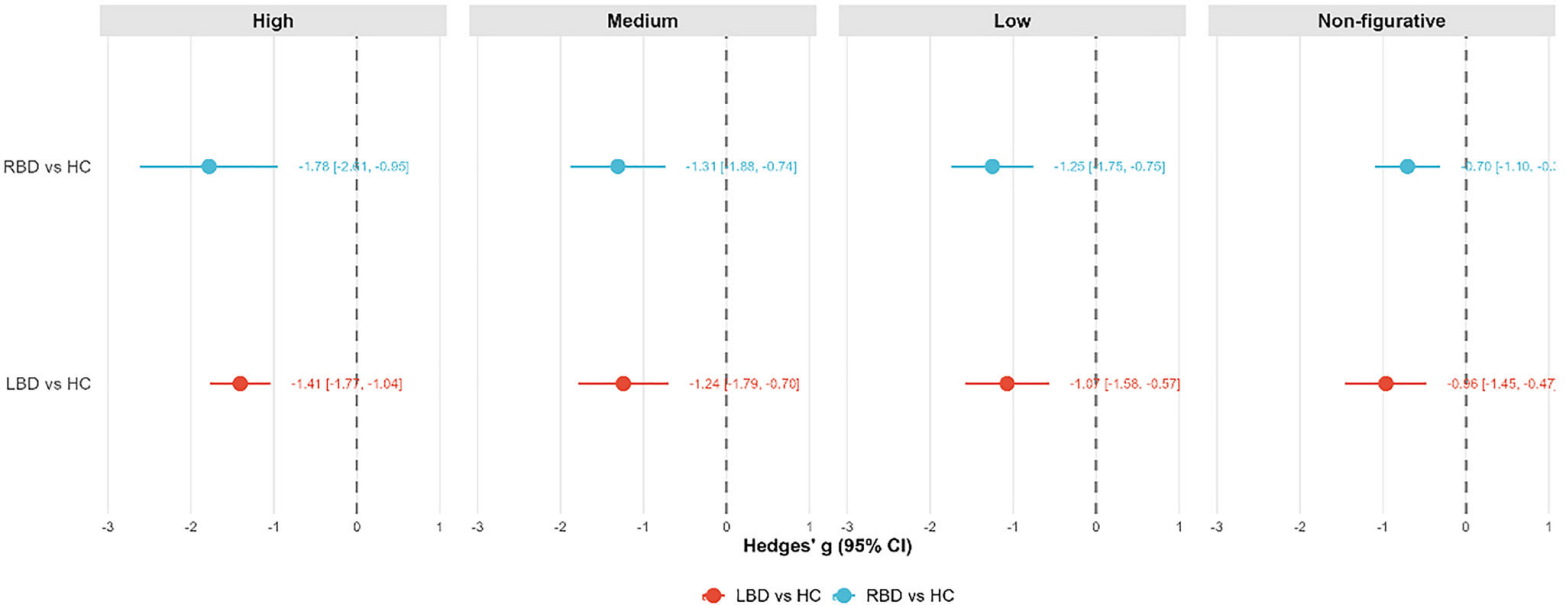
Impairment patterns of LBD and RBD patients across conventionality levels (random‐effects model). (LBD = left‐brain‐damage patients; RBD = right‐brain‐damage patients; HC = healthy controls).

#### 3.2.1 LBD Patients Versus Healthy Controls

For LBD patients, significant deficits relative to healthy controls were observed across all conventionality levels. The largest effect size emerged for high‐conventionality items (Hedges’ *g* = −1.41, 95% CI [−1.77, −1.04], *p* < 0.001, *k* = 35, *I* ^2^ = 73.3%), indicating severe impairment in processing conventionalized figurative expressions. Medium‐conventionality items also showed a substantial deficit (Hedges’ *g* = −1.24, 95% CI [−1.79, −0.70], *p* = .001, *k* = 11, *I* ^2^ = 60.4%), as did low‐conventionality items (Hedges’ *g* = −1.07, 95% CI [−1.58, −0.57], *p* = 0.001, *k* = 13, *I* ^2^ = 74.1%). The non‐figurative/literal baseline condition revealed a significant but somewhat smaller deficit (Hedges’ *g* = −0.96, 95% CI [−1.45, ‐0.47], *p* = 0.002, *k* = 11, *I* ^2^ = 55.9%).

#### 3.2.2 RBD Patients Versus Healthy Controls

RBD patients also demonstrated significant impairments across all conventionality levels. The largest effect size was again observed for high‐conventionality items (Hedges’ *g* = −1.78, 95% CI [−2.61, −0.95], *p* < 0.001, *k* = 16, *I* ^2^ = 86.5%), with numerically larger deficits than those seen in LBD patients. Medium‐conventionality items showed a substantial deficit (Hedges’ *g* = −1.31, 95% CI [−1.88, −0.74], *p* < 0.001, *k* = 16, *I* ^2^ = 78.2%), as did low‐conventionality items (Hedges’ *g* = −1.25, 95% CI [−1.75, −0.75], *p* < 0.001, *k* = 19, *I* ^2^ = 84.8%). The non‐figurative baseline condition revealed a significant but smaller deficit (Hedges’ *g* = −0.70, 95% CI [−1.10, −0.31], *p* = 0.002, *k* = 17, *I* ^2^ = 69.0%). In total, these analyses included 1135 RBD patients and 1291 healthy controls across all conventionality levels.

## 4 Discussion

### 4.1 Hemispheric Contributions to Figurative Language Comprehension: Insights From the Direct Comparisons

The direct comparisons between LBD and RBD patients revealed no significant overall difference in figurative language comprehension, suggesting that damage to either hemisphere can impair the ability to understand figurative language. Although the interaction between conventionality and lesion side did not reach statistical significance, the pattern of effect sizes across conventionality levels revealed meaningful trends that align with theoretical predictions. For high‐conventionality items, LBD patients showed a trend toward greater deficits than RBD patients (Hedges’ *g* = −0.242); for low‐conventionality items, the opposite trend emerged, with RBD patients showing numerically larger deficits (Hedges’ *g* = 0.225, *p* = 0.086). This pattern suggests that hemispheric contributions to figurative language may shift as a function of conventionality, with the left hemisphere playing a more prominent role in processing conventionalized expressions and the right hemisphere becoming increasingly engaged as expressions become more novel. Besides, the non‐figurative baseline condition revealed that LBD patients performed significantly worse than RBD patients, indicating that left‐hemisphere damage has a greater impact on literal language processing. This finding is consistent with the established role of the left hemisphere in fine semantic processing (Jung‐Beeman 2005) and serves as an important validation of our patient samples: The expected left‐hemisphere dominance for literal language was indeed observed.

These trends are consistent with the theoretical frameworks discussed in the Introduction section. According to the career of metaphor (Bowdle and Gentner 2005; Wolff and Gentner 2011), highly conventionalized expressions such as idioms and proverbs are stored and retrieved as lexicalized units, engaging left‐hemisphere semantic memory networks. In contrast, low‐conventionality expressions require the right hemisphere to support complex computational processes—including inference, structural mapping, and analogical reasoning—that are essential for novel figurative meaning construction (Gentner and Wolff 1997; Wolff and Gentner 2011). The fine‐coarse semantic coding theory (Jung‐Beeman 2005) further proposes that the left hemisphere specializes in fine semantic coding (focused activation of closely related meanings), while the right hemisphere engages in coarse semantic coding (broader activation of distantly related concepts), making it particularly suited for integrating information across distant conceptual domains. The observed trends—LBD patients showing numerically larger deficits for high‐conventionality items and RBD patients showing numerically larger deficits for low‐conventionality items—provide empirical support for these theoretical predictions in the context of brain damage.

The indirect comparisons with healthy controls provide important context for interpreting these trends.^4^ Both patient groups showed significant deficits relative to controls across all conventionality levels, confirming that unilateral brain damage to either hemisphere compromises figurative language comprehension. Notably, RBD patients exhibited numerically larger deficits than LBD patients across all figurative language types (high: −1.78 vs. −1.41; medium: −1.31 vs. −1.24; low: −1.25 vs. −1.07), suggesting that right‐hemisphere damage may lead to more severe relative impairments in figurative language processing compared to healthy controls. This pattern appears to conflict with the direct comparison trends, which suggested that LBD patients were relatively worse for high‐conventionality items.

However, this apparent discrepancy can be resolved by considering that healthy control performance is not uniform across conventionality levels. Healthy adults typically perform better on high‐conventionality items than on low‐conventionality items, as conventionalized expressions are more familiar, more predictable, and require less cognitive effort to process (Lai et al. 2009; Tang et al. 2017). When this baseline variation in healthy control performance is taken into account, the pattern of LBD patients’ performance becomes particularly informative. Although LBD patients’ deficits decrease numerically across conventionality levels (from −1.41 to −1.07), this decrease is comparable to—or even slightly smaller than—the expected decrease in healthy control performance. This suggests that LBD patients’ capacity to comprehend figurative language may be relatively stable across conventionality levels when the inherent difficulty of the items is accounted for. In other words, despite left‐hemisphere damage that compromises their ability to process language generally, LBD patients appear to engage their intact right hemispheres to support the complex computational processes required for understanding less conventionalized figurative expressions.

For LBD patients with intact right hemispheres, the cognitive mechanisms described above—structural mapping (Gentner and Wolff 1997; Wolff and Gentner 2011), analogical reasoning, and coarse semantic coding (Jung‐Beeman 2005)—remain available, enabling them to perform on low‐conventionality items at a level comparable to their performance on high‐conventionality items when baseline difficulty supported by performance of healthy controls is accounted for. In contrast, RBD patients, despite intact left‐hemisphere specialization for conventionalized language, show a steeper performance decline as conventionality decreases, consistent with the loss of right‐hemisphere resources crucial for novel figurative meaning construction. Thus, while both patient groups show decreasing relative deficits compared to healthy controls as conventionality decreases, the underlying interpretations differ fundamentally. For RBD patients, this decrease likely reflects the increasing difficulty of low‐conventionality items for healthy controls, masking what may be genuine deficits in processing novel figurative language. For LBD patients, the relatively stable performance across conventionality levels—when considered against the backdrop of healthy control performance—provides evidence for the right hemisphere's critical contribution to the cognitive operations underlying figurative language comprehension. This interpretation is consistent with the direct comparison trends, which show LBD patients performing relatively better than RBD patients on low‐conventionality items—a pattern that directly supports the hypothesized right‐hemisphere specialization for novel figurative language processing.

Several caveats merit consideration when interpreting these findings. First, the patterns observed in the direct comparisons were trends rather than statistically significant effects for most conventionality levels, reflecting the substantial heterogeneity across studies and the relatively small number of studies in some subgroups. Second, the theoretical frameworks discussed—the career of metaphor (Bowdle and Gentner 2005; Wolff and Gentner 2011), the structure mapping theory (Gentner and Markman 1997; Wolff and Gentner 2011), and the fine‐coarse semantic coding theory (Jung‐Beeman 2005)—were developed primarily based on studies with healthy adults. Their application to brain‐damaged populations requires caution, as lesion studies reveal not only the functional contributions of damaged regions but also the compensatory potential of intact areas. The deficits observed in LBD and RBD patients reflect a combination of functional loss in the damaged hemisphere and potential reorganization in the unaffected hemisphere, a distinction that cannot be fully disentangled in the present meta‐analysis. Third, the processing of low‐conventionality items likely involves bilateral cooperation rather than exclusive right‐hemisphere engagement (Mashal et al. 2007), consistent with the substantial deficits observed in both patient groups relative to controls.

In summary, the direct comparisons, complemented by the indirect comparisons with healthy controls, provide partial support for the career of metaphor (Bowdle and Gentner 2005), the structure mapping theory (Gentner and Markman 1997; Wolff and Gentner 2011), and the fine‐coarse semantic coding theory (Jung‐Beeman 2005) in the context of brain damage. High‐conventionality expressions appear more dependent on left‐hemisphere integrity, while low‐conventionality expressions show greater vulnerability to right‐hemisphere damage. These findings advance our understanding of how hemispheric contributions to figurative language are shaped by conventionality, while also highlighting the need for future research to disentangle functional loss from neuroplastic reorganization.

### 4.2 Moderating Factors Influencing Hemispheric Differences

The moderator analysis of LBD subtype showed that the presence of aphasia did not significantly moderate the overall LBD‐RBD difference. This null finding suggests that the presence or absence of aphasia does not systematically alter the pattern of hemispheric differences in figurative language comprehension. However, this result should be interpreted with caution. The included studies varied considerably in how aphasia was characterized, and most did not provide detailed information on aphasia subtypes or severity. Moreover, all included studies required that patients retain sufficient comprehension to understand task instructions, meaning that even the aphasic patients in these samples had at least basic receptive language abilities. Thus, the lack of a significant moderating effect of aphasia status does not necessarily imply that aphasia is irrelevant to figurative language comprehension; rather, it may reflect the heterogeneity of aphasia definitions and the relatively coarse distinction between “aphasic” and “non‐aphasic” groups used in the original studies. Future research with more fine‐grained characterization of aphasia subtypes and severity is needed to clarify whether specific language profiles differentially affect figurative language processing.

In contrast to LBD subtype, sample size significantly moderated the LBD‐RBD effect sizes. Specifically, smaller studies tended to yield more extreme effect estimates—either showing a larger LBD disadvantage or a larger LBD advantage relative to RBD patients—whereas larger studies produced effect sizes closer to zero. This pattern suggests that smaller studies may be more susceptible to random error or selection bias, leading to estimates that deviate from the true population effect (Cuijpers et al. 2021).

Task type also significantly moderated the overall LBD‐RBD difference. Specifically, LBD patients showed significantly poorer performance than RBD patients in verbal meaning explanation tasks, whereas no significant differences were observed in multiple picture selection, multiple sentence/word selection, or yes–no question tasks.

This pattern suggests that LBD patients’ difficulties, regardless of aphasia status, may be more pronounced in language production than in language comprehension per se, as verbal meaning explanation tasks place heavy demands on lexical retrieval and verbal formulation, which are known to rely on left‐hemisphere networks (Friederici and Alter 2004). When these production demands are minimized, as in picture selection or multiple‐choice tasks, the performance difference between LBD and RBD patients diminishes or disappears. This interpretation is supported by the absence of significant LBD‐RBD differences in the other three task types—if LBD patients had a fundamental impairment in figurative language comprehension, such a deficit would likely manifest across different task formats, rather than only in tasks requiring verbal output.

Nevertheless, this inference should be interpreted with caution. Given that most studies did not report performance separately for fluent versus non‐fluent aphasic patients, we are currently unable to directly test the production‐difficulty account. Future research should report aphasia subtype and performance across different task conditions in greater detail, in order to more precisely disentangle the respective roles of comprehension deficits and production difficulties in figurative language processing.

This finding has implications for clinical assessment: Verbal meaning explanation tasks may underestimate the actual comprehension abilities of patients with expressive difficulties. Therefore, clinical assessment should adopt a multi‐modal approach, combining both production and comprehension tasks, to more accurately evaluate patients' true capacities and to avoid underestimation or overestimation due to inappropriate task selection.

Number of trials did not significantly moderate the overall effect, indicating that task length did not systematically influence hemispheric differences within the range of trial numbers represented in the included studies.

In summary, the moderator analyses reveal that methodological factors—particularly sample size and task type—can influence the observed pattern of hemispheric differences in figurative language comprehension. Sample size moderated the overall effect, suggesting that smaller studies tend to produce more extreme estimates. Task type also moderated the overall effect, with verbal meaning explanation tasks being particularly sensitive to left‐hemisphere damage. In contrast, LBD subtype and number of trials did not significantly moderate the overall effect. These findings highlight the importance of considering methodological factors when designing and interpreting studies on figurative language comprehension in brain‐damaged populations.

### 4.3 Implications

The conventionality‐dependent pattern of hemispheric contributions provides empirical support for the career of metaphor (Bowdle and Gentner 2005) in brain‐damaged populations: high‐conventionality expressions rely more on left‐hemisphere mechanisms, whereas low‐conventionality expressions show greater vulnerability to right‐hemisphere damage. This pattern, while not reaching statistical significance as an interaction, is consistent with theoretical predictions and is further supported by the indirect comparisons when healthy control baseline performance is taken into account. The findings also align with the fine‐coarse semantic coding theory (Jung‐Beeman 2005) and the structural mapping processes described by Gentner and Wolff (1997), though the significant deficits in both groups for low‐conventionality items indicate bilateral cooperation rather than exclusive right‐hemisphere engagement.

The task type findings have direct implications for the design of clinical assessments. The significant LBD disadvantage in verbal meaning explanation tasks, but not in other task formats, suggests that tasks requiring explicit verbal production may underestimate the figurative language comprehension abilities of patients with expressive difficulties. Conversely, tasks that minimize production demands (e.g., picture selection or multiple‐choice formats) may provide a more accurate reflection of comprehension abilities. These findings are consistent with Lada et al. (2023), who highlighted that task type—whether string‐to‐picture matching, string‐to‐word matching, or oral definition—can significantly influence patients’ performance patterns. Taken together, these considerations suggest that no single task format is universally appropriate; rather, assessment should combine multiple task types to obtain a comprehensive evaluation of a patient's abilities.

The significant moderating effect of sample size serves as a methodological caution: Smaller studies tend to yield more extreme effect estimates. This underscores the need for adequately powered studies in this field and suggests that meta‐analytic findings should be interpreted with consideration of the sample sizes of contributing studies.

### 4.4 Limitations

Several limitations should be acknowledged. First, the number of studies in certain subgroups was limited (e.g., low‐conventionality: *k* = 8), restricting our ability to detect statistically significant effects and conduct fine‐grained moderator analyses. Second, substantial heterogeneity remained unexplained even after accounting for the moderators examined, reflecting unmeasured factors such as specific lesion locations, time post‐onset, and subtle differences in stimulus materials. Third, demographic and clinical characteristics—particularly lesion size, lesion site, aphasia severity, and aphasia subtype—were inconsistently reported across studies, precluding their systematic inclusion as moderators in the present meta‐analysis. While we were able to characterize these variables descriptively (see Table 3 and Section 3.1.1), the heterogeneity in reporting formats prevented quantitative analyses. This limitation is particularly relevant for interpreting the null finding of LBD subtype, as the coarse distinction between “aphasic” and “non‐aphasic” may have masked important heterogeneity in aphasia profiles. Fourth, all included studies used offline accuracy measures; the small number of studies reporting online measures (e.g., reaction times, ERP) precluded their inclusion, leaving questions about processing dynamics unanswered. Fifth, the theoretical frameworks invoked were developed primarily from healthy adults; their application to brain‐damaged populations requires caution, as lesion studies reveal not only functional loss but also compensatory reorganization.

Future research should address these limitations by systematically reporting detailed patient characteristics (lesion location; lesion size; aphasia type and severity), combining lesion mapping with functional neuroimaging to directly test hypotheses about compensatory reorganization, employing online measures to track real‐time processing, and using well‐powered, multi‐center designs to obtain reliable effect size estimates in this heterogeneous population.

## 5 Conclusion

This meta‐analysis provides the first quantitative analysis of hemispheric contributions to figurative language comprehension in patients with left‐ and right‐hemisphere damage. Although the interaction between lesion side and conventionality did not reach statistical significance, the observed trends across conventionality levels align with theoretical predictions: High‐conventionality expressions tend to rely more on the left‐hemisphere, while low‐conventionality expressions show a trend toward greater vulnerability to right‐hemisphere damage. Indirect comparisons confirmed that both patient groups are significantly impaired relative to healthy controls. These results are broadly consistent with the career of metaphor (Bowdle and Gentner 2005) and the fine‐coarse semantic coding theory (Jung‐Beeman 2005), while also highlighting that methodological factors, particularly task type, can influence the observed pattern of hemispheric differences. The findings suggest that assessments of figurative language comprehension should consider both conventionality and task demands, as performance on one type of task may not generalize to another.

## Author Contributions

**Lexian Shen**: conceptualization, data curation, formal analysis, investigation, methodology, project administration, software, visualization, writing – original draft, writing – review and editing. **Man Fu**: data curation, formal analysis, investigation, writing – original draft, writing – review and editing. **Min Wang**: funding acquisition, writing – original draft, writing – review and editing. **Xuemei Tang**: writing – review and editing, supervision. **Hui Chang**: supervision, writing – review and editing, methodology, project administration, resources.

## Funding

This study was funded by the Youth Project of the Anhui Provincial Philosophy and Social Science Planning Program (Grant award number AHSKYQ2025D53).

## Disclosure

During the preparation of this work, the author(s) used DeepSeek (DeepSeek‐V3) to assist with English language editing and to improve the clarity and readability of the manuscript. This use did not impact the key arguments or conclusions of the study. The authors provided all scientific content, including study design, data analysis, theoretical interpretations, and core arguments. After using this tool, the author(s) carefully reviewed and edited the content as needed and take full responsibility for the final content of the published article.

## Ethics Statement

The authors have nothing to report.

## Conflicts of Interest

The authors declare no conflicts of interest.

## Supporting information



Table S1. Detailed aphasia‐related characteristics of LBD groups in studies included in the direct LBD‐RBD comparison.

## Data Availability

The data supporting the findings of this meta‐analysis have been deposited in the Open Science Framework (OSF) repository and will be made publicly available upon publication. During the review process, data can be accessed via the private link: [https://osf.io/xth67/overview?view_only=75462cb0bd4d4c74a4ccdb4c4642c694].

## Endnotes

1 Given that only 20 studies from eight articles reported data of reaction time or reading time, and these were distributed across only 2–3 studies per conventionality level, these data were not included in the present meta‐analysis (e.g., Milburn et al. 2018).

2 We initially aimed to examine the effect of lesion region (anterior vs. posterior) based on the findings of Winner and Gardner (1977), who reported differential metaphor comprehension deficits between Broca's (anterior) and Wernicke's (posterior) aphasia. However, the majority of included studies did not provide sufficient anatomical detail to reliably classify patients into anterior and posterior subgroups. Therefore, this variable was not included in the meta‐analysis.

3 Three articles included in the direct comparisons did not contribute to the indirect comparisons because they either did not include a healthy control group (Brownell et al. 1990; Kempler et al. 1999) or did not report sufficient raw data or statistic results of healthy controls (Winner and Gardner 1977). In addition, six articles (Brownell et al. 1983; Giora et al. 2000; Marulanda‐Páez and Igoa‐González 2021; Papagno et al. 2004; Sidtis and Yang 2017; Tsolakopoulos et al. 2023) reported only inferential statistics for patient–control comparisons; however, the Hedges’ *g* values calculated from these statistics were identified as outliers (exceeding 2.5 standard deviations from the mean) in subsequent meta‐analytic diagnostics and were therefore excluded from the indirect comparisons.

4 The direct comparisons benefit from greater methodological consistency, as they compare LBD and RBD patients within the same studies using identical stimuli and procedures. This makes direct comparisons more reliable for assessing hemispheric differences than comparisons derived from separate study samples. The indirect comparisons, while valuable for establishing that both patient groups are impaired relative to healthy controls, involve different study samples for each patient group and should be interpreted with caution when making cross‐group comparisons.
