# Supplementary material for: Hemispheric Contributions to Figurative Language Comprehension After Unilateral Brain Damage: A Meta‐Analysis of Lesion Side and Conventionality Interaction
Source: Brain Behav. 2026 Oct 7;16(10):e71828. doi: 10.1002/brb3.71828 (PMC13646486; doi:10.1002/brb3.71828)
Supplement: Supplementary file 1 [file BRB3-16-e71828-s001.docx]

Table S1. Detailed aphasia-related characteristics of LBD groups in studies included in the direct LBD-RBD comparison.

| Study | Aphasia state | Aphasia severity | Aphasia subtype |
| --- | --- | --- | --- |
| Aydin et al. (2017) | Aphasia | GAT-2 ≥ 75% | Not reported |
| Cardillo et al. (2018) | Nonaphasia | / | / |
| Zaidel et al. (2002) | Aphasia | WAB-based classification | Global, Broca, Wernicke, Conduction, Anomic, and Unclassified |
| Giora et al. (2000) | Aphasia | WAB-based classification | Global, Broca, Wernicke, Conduction, Anomic, and Unclassified |
| Kempler et al. (1999) | Aphasia | Not reported | Not reported |
| Brundage (1993) | Aphasia | SPICA percentile = 72 ± 10.0 | Nonfluent = 3, Fluent-mixed = 3, Anomic = 4 |
| Brownell et al. (1990) | Aphasia | Not reported | Anterior (nonfluent) = 6, Posterior (fluent) = 8, Mixed = 5 |
| Bryan (1988) | Aphasia | WAB AQ = 63.8±19.7 | Not reported |
| England (1986) | Aphasia | PICA Overall=13.30 | Not reported |
| Winner and Gardner (1977) | Aphasia | Not reported | Broca = 4, Wernicke = 7, Unclassified = 24 |
| IITAKA et al. (2003) | Aphasia | SLTA ≥40% | Fluent = 17, Nonfluent = 10 |
| Murphy et al. (2013) | Nonaphasia | / | / |
| Shamay-Tsoory et al. (2005) | Nonaphasia | / | / |

Note: RBD groups were nonaphasic in all studies and are not listed separately.
